# Three-Domain Serial Cranial Ultrasound Phenotypes and Outcomes in Very Preterm Infants with Severe Brain Injury: A Single-Center Cohort Study

**DOI:** 10.3390/children13070844

**Published:** 2026-06-23

**Authors:** Noemí Núñez-Enamorado, Ana Camacho-Salas, María López-Maestro, María Carmen Gallego-Herrero, Ana Martínez de Aragón, Sara Vila-Bedmar, Sara Vázquez-Román, Berta Zamora-Crespo, Carmen Rosa Pallás-Alonso, María Teresa Moral-Pumarega

**Affiliations:** 1 Division of Pediatric Neurology, Hospital Universitario 12 de Octubre, Avenida de Córdoba s/n, 28041 Madrid, Spain; acamacho@salud.madrid.org (A.C.-S.); sara.vila@salud.madrid.org (S.V.-B.); 2 Program in Research in Medical and Surgical Sciences, Faculty of Medicine, Complutense University of Madrid, 28040 Madrid, Spain; carmenrosa.pallas@salud.madrid.org (C.R.P.-A.); mmoralp@salud.madrid.org (M.T.M.-P.); 3 Neonatology Unit, Hospital Universitario 12 de Octubre, Avenida de Córdoba s/n, 28041 Madrid, Spain; mlmaestro@salud.madrid.org (M.L.-M.); sara.vazquez@salud.madrid.org (S.V.-R.); 4 Division of Pediatric Radiology, Hospital Universitario 12 de Octubre, Avenida de Córdoba s/n, 28041 Madrid, Spain; cgallego@salud.madrid.org (M.C.G.-H.); amaragoncalvo@salud.madrid.org (A.M.d.A.); 5 Division of Neuropsychology, Hospital Universitario 12 de Octubre, Avenida de Córdoba s/n, 28041 Madrid, Spain; berta.zamora@salud.madrid.org

**Keywords:** neonatal brain injury, prematurity-related brain injury, neurodevelopmental outcomes, neuroimaging, cranial ultrasound, very preterm infants, periventricular hemorrhagic infarction, intraventricular hemorrhage, cystic periventricular leukomalacia, withdrawal of life-sustaining treatment

## Abstract

**Highlights:**

**What are the main findings?**

- A structured serial cranial ultrasound framework combining three mutually exclusive severe brain injury entities, periventricular hemorrhagic infarction (PVHI), the grade 3 intraventricular hemorrhage entity (IVH3 entity), and cystic periventricular leukomalacia (cPVL), with three imaging domains, parenchymal lesion, intraventricular hemorrhage, and ventriculomegaly, described distinct entity-specific trajectories.
- Using a common maximal-burden cranial ultrasound summary, deaths following documented treatment limitation for poor neurological prognosis occurred in infants with greater multidomain imaging burden than survivors, while survivor outcomes differed by lesion entity, motor phenotype, and functional severity.

**What is the implication of the main finding?**

- Serial cranial ultrasound may support a structured, transparent, cohort-level description of severe brain injury trajectories by separating lesion entity, documented timing and multidomain imaging burden.
- Severe multidomain imaging burden was associated with higher outcome burden, but should not be interpreted as a deterministic prognostic label or as a decision rule, because survivor outcomes vary by lesion entity and functional severity.

**Abstract:**

**Background/Objectives:** Severe brain injury (SBI) in very preterm infants includes heterogeneous lesions with distinct timing, burden and outcomes. We used cranial ultrasound (CUS) to describe SBI entity, documented timing, three-domain burden, deaths following documented withdrawal, withholding or non-escalation of life-sustaining treatment for poor neurological prognosis (neuro-WWLST), and survivor outcomes. **Methods:** Retrospective single-center cohort (1991–2020) of 2841 very preterm infants (<32 weeks’ gestation and/or birth weight ≤ 1500 g) with complete CUS within 48 h after birth. CUS was summarized by four windows, three domains—parenchymal lesion, intraventricular hemorrhage (IVH) and ventriculomegaly—and three mutually exclusive entities: periventricular hemorrhagic infarction (PVHI), cystic periventricular leukomalacia (cPVL and grade 3 IVH without PVHI/cPVL (IVH3 entity). Cross-outcome analyses used common maximal-burden CUS. **Results:** SBI occurred in 286/2841 infants (10.1%) and neuro-WWLST death in 45/2841 infants (1.6%); 43/45 occurred within SBI, and 43/89 SBI deaths (48.3%) followed documented neuro-WWLST. Using common maximal-burden CUS, severe three-domain involvement was more frequent among neuro-WWLST deaths than survivors (37.2% vs. 8.6%). Among SBI survivors with follow-up, cerebral palsy (CP) occurred in 87/176 (49.4%) and clinically classified school-age cognitive sequelae in 50/155 (32.3%). Outcomes varied by entity, with mainly ambulatory unilateral CP after PVHI, more frequent non-ambulatory bilateral CP after cPVL, and a heterogeneous IVH3 profile. Severe three-domain involvement identified a small subgroup with higher outcome burden, but outcomes were not deterministic. **Conclusions:** A structured, descriptive CUS approach separating lesion entity, documented timing and multidomain burden may support transparent cohort-level description of SBI trajectories, documented neuro-WWLST deaths and survivor outcomes.

## 1. Introduction

In very preterm infants, severe brain injury (SBI) encompasses several major lesion types, including periventricular hemorrhagic infarction (PVHI), grade 3 intraventricular hemorrhage (IVH), and cystic periventricular leukomalacia (cPVL). These lesions remain important contributors to neonatal death and later neurodevelopmental impairment [1,2,3,4,5], and contemporary cohorts continue to report a substantial SBI burden [6,7,8]. Among survivors, the risk of cerebral palsy (CP), cognitive impairment, and other neurological sequelae varies according to lesion type, extent, ventricular complications, and coexisting brain injury, rather than by lesion label alone [1,2,9,10,11,12,13,14,15,16]. Some deaths after SBI occur following withdrawal or withholding of life-sustaining treatment for neuroprognostic reasons (neuro-WWLST), although reported frequencies vary across settings [17,18,19,20,21,22].

Cranial ultrasound (CUS) is the main bedside neuroimaging modality in neonatal intensive care because it identifies major lesions, can be repeated in clinically unstable infants, and allows for serial assessment of lesion evolution. Current guidance emphasizes appropriately timed and descriptively reported neonatal CUS, as serial bedside imaging provides diagnostic and prognostic information during neonatal care [23,24]. Previous serial CUS studies have substantially advanced the description of preterm brain injury, including PVHI, germinal matrix–intraventricular hemorrhage, posthemorrhagic ventricular dilatation, white matter injury, and their relationships with outcome [9,10,16,23,24,25]. In contemporary practice, MRI provides complementary information on lesion extent, brain maturation, and subtle parenchymal injury, particularly around term-equivalent age [26,27,28]. However, CUS remains the repeated bedside modality available across unstable early NICU courses and across historical cohorts.

Within this clinical setting, many cohort reports still summarize SBI primarily by lesion category, hemorrhage grade, mortality, or final outcome, whereas serial CUS can additionally describe when the lesion becomes classifiable, whether imaging burden accumulates, and whether parenchymal injury, IVH, and ventriculomegaly (VM) evolve together or separately.

This distinction is important because SBI entities follow different temporal and prognostic patterns. PVHI and IVH3 usually become classifiable early, whereas cPVL often becomes clearly cystic only on later scans [6,9,23]. PVHI is also heterogeneous in topography and course, including associated IVH and evolving VM or posthemorrhagic hydrocephalus [10,11,12,13,14]. Moreover, neonatal CUS markers of white matter injury and ventricular enlargement have been associated with later neurodevelopmental impairment, supporting the clinical relevance of considering lesion burden across more than one imaging domain [16,29,30,31,32]. Previous studies have linked severe IVH, PVHI, and major brain injury with treatment limitation, regional variation in mortality, or WWLST discussions [2,14,20,22,33]. Nevertheless, fewer studies have combined mutually exclusive SBI entities, prespecified serial CUS windows, multidomain ultrasound burden, and outcome-group comparisons distinguishing survivors, deaths not classified as neuro-WWLST, and deaths following documented neuro-WWLST. The serial CUS phenotype and outcome-proximal bedside imaging context of deaths following documented neuro-WWLST therefore remain insufficiently characterized.

We asked whether routinely acquired serial CUS could be organized into a clinically interpretable framework for describing SBI trajectories in a single-center cohort of very preterm infants (<32 weeks’ gestational age (GA) and/or birth weight (BW) ≤ 1500 g). The primary objective was to characterize three mutually exclusive SBI entities (PVHI, the IVH3 entity, and cPVL) by timing of documented classifiability across four prespecified CUS windows and by burden across three imaging domains: parenchymal lesion, IVH, and VM. The secondary objective was to compare in-hospital outcome groups—survivors, deaths not classified as neuro-WWLST, and deaths following documented neuro-WWLST—using a common maximal-burden CUS definition applied uniformly across all groups. Among infants whose deaths followed documented neuro-WWLST, we additionally described last-window CUS as a separate outcome-proximal imaging summary. As an exploratory clinical extension, we described cerebral palsy and clinically classified school-age cognitive sequelae among survivors according to the number of severe imaging domains and assessed whether severe involvement across all three domains was associated with these outcomes.

This approach was intended to describe serial imaging phenotypes, maximal documented imaging burden, and outcome-proximal imaging context; it was not designed to evaluate CUS as a decision rule, prognostic score, or causal model of outcome.

## 2. Materials and Methods

### 2.1. Study Design and Cohort

This retrospective cohort study used a prospectively maintained single-center tertiary NICU registry from 1991 to 2020. Eligible infants were born at <32 weeks’ GA and/or had BW ≤ 1500 g. Imaging-based analyses were restricted to infants with a complete recorded CUS1 assessment within 48 h after birth. In-hospital outcomes were assessed until discharge or death. Among survivors, neurodevelopmental follow-up included CP assessment at approximately 2 years’ corrected age and clinically classified school-age cognitive outcome around the routine 7-year follow-up window. Death following documented neuro-WWLST was defined as in-hospital death following documented withdrawal, withholding, or non-escalation of life-sustaining treatment primarily because of poor neurological prognosis, as recorded in the neonatal unit registry and discharge/death summaries. The registry captured neuro-WWLST only when this process culminated in death; it did not systematically capture counseling about poor neurological prognosis, treatment-limitation discussions, decisions not followed by death, or non-neurologic treatment-limitation pathways. Additional methodological details on cohort assembly, CUS-window definitions, MRI availability, available registry data, neuro-WWLST ascertainment, scan-selection rules, missing data, and neurodevelopmental follow-up procedures are provided in Supplementary Methods S1–S7. This study is reported in accordance with the STROBE recommendations for observational cohort studies; the completed checklist is provided in the Supplementary Table S27.

### 2.2. Serial CUS Framework and SBI Entities

Serial CUS was summarized within four prespecified windows: ≤48 h (CUS1), around day 7 (CUS2), around day 28 (CUS3), and term-equivalent age or discharge, whichever occurred first (CUS4). CUS examinations were performed and reported as part of routine clinical care by the pediatric radiology team, including senior pediatric radiologists. The reported findings were prospectively entered into the neonatal registry and constituted the CUS data source for this study. Stored images were not systematically re-reviewed, and formal interobserver reliability was not assessed. For each prespecified CUS window, parenchymal lesion, IVH, and VM were analyzed as separate imaging domains. Severe imaging domains were prespecified as parenchymal lesion ≥ 1 cm, grade 3 IVH, and moderate-to-severe VM (VM ≥ 2). Severe involvement across all three domains denoted concurrent presence of all three findings. SBI was defined a priori as three mutually exclusive entities: periventricular hemorrhagic infarction (PVHI), cystic periventricular leukomalacia (cPVL), and the IVH3 entity. PVHI denoted a periventricular hemorrhagic parenchymal lesion on serial CUS, irrespective of associated IVH grade. cPVL denoted cystic periventricular white matter injury. The IVH3 entity comprised infants reaching grade 3 IVH during follow-up without meeting PVHI or cPVL criteria, including isolated grade 3 IVH and grade 3 IVH, with additional parenchymal injury not fulfilling PVHI or cPVL criteria. Entity debut was the first CUS window meeting entity criteria. Detailed grading and mutual-exclusivity rules are provided in Supplementary Methods S3–S4.

### 2.3. Outcomes and Scan Definitions

In-hospital outcomes were categorized as survival to discharge, death not classified as neuro-WWLST in the registry, and death following documented neuro-WWLST. For the primary cross-outcome imaging comparison, one maximal-burden CUS was selected for each infant using the same prespecified hierarchical rule across all three outcome groups. The scan-selection rule and associated robustness and sensitivity analyses are detailed in Supplementary Methods S6. Among deaths following documented neuro-WWLST, last-window CUS, defined as the last recorded CUS before death within the prespecified surveillance windows, was additionally summarized to describe the outcome-proximal imaging context.

Neurodevelopmental outcomes were analyzed exploratorily among survivors. Cerebral palsy (CP) was assessed at approximately 2 years’ corrected age and classified by Gross Motor Function Classification System (GMFCS) level. Clinically classified school-age cognitive sequelae were categorized as absent, mild, moderate or severe using available longitudinal follow-up information, usually around the 7-year assessment window. Cognitive outcomes were not analyzed as harmonized continuous IQ endpoints, and these analyses were considered exploratory. Full definitions and follow-up procedures are provided in Supplementary Methods S7.

### 2.4. Statistical Analysis

Continuous variables are reported as median [IQR] and categorical variables as n/N (%). The primary cross-outcome imaging comparison used the common maximal-burden CUS definition across all in-hospital outcome groups. Pairwise descriptive comparisons contrasted deaths following documented neuro-WWLST with survivors and with deaths not classified as neuro-WWLST. Continuous and ordinal variables were compared using Mann–Whitney U tests, and categorical variables using Fisher’s exact test. Effect sizes were reported as Hodges–Lehmann shifts or unadjusted odds ratios with 95% confidence intervals. These estimates were descriptive and were not used for prediction or causal inference. Exploratory survivor outcome analyses compared infants with severe involvement across all three domains with those with fewer than three severe domains. For CP and clinically classified school-age cognitive sequelae, Fisher’s exact tests were followed by exploratory logistic regression models adjusted for GA, birth epoch, and SBI entity. Selected sensitivity models replaced GA with BW while retaining birth epoch and SBI entity, because GA and BW were not entered together given collinearity and sparse event counts. These adjusted models were interpreted cautiously because of small subgroup sizes and heterogeneous ascertainment of cognitive outcomes; they were not used for prediction. No multiplicity adjustment was applied. Scan-selection robustness and sensitivity analyses are described in Supplementary Methods S6. Analyses were performed using Stata/SE 18.0 (StataCorp LLC, College Station, TX, USA).

### 2.5. Use of Generative Artificial Intelligence Tools

Generative artificial intelligence tools were used for language editing and formatting support during manuscript preparation. All scientific content, data analyses, interpretations, and final wordings were reviewed and approved by the authors. No generative artificial intelligence tool was used to analyze the study dataset.

## 3. Results

### 3.1. Cohort, SBI Burden and Epoch Patterns

**Figure 1:**
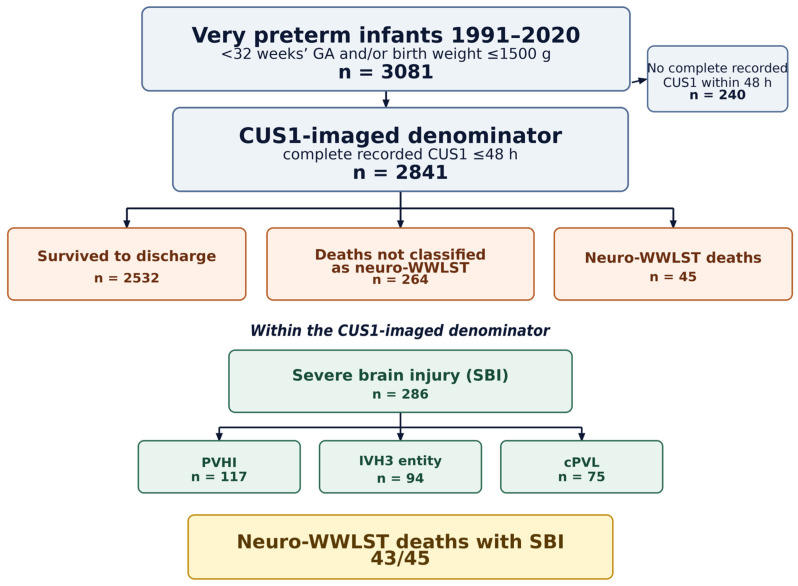
Study cohort, in-hospital outcomes, and severe brain injury entities. The CUS1-imaged denominator comprised infants with a complete recorded CUS1 assessment within 48 h after birth. Arrows indicate cohort flow; box colors distinguish the cohort denominator, in-hospital outcome categories, SBI entities, and neuro-WWLST deaths with SBI. In-hospital outcomes were survival to discharge, death not classified as neuro-WWLST in the registry, and death following documented neuro-WWLST. SBI comprised three mutually exclusive entities: PVHI, the IVH3 entity, and cPVL. PVHI was classified irrespective of coexisting IVH grade. The IVH3 entity comprised infants reaching grade 3 IVH without meeting PVHI or cPVL criteria. Neuro-WWLST deaths with SBI denotes the proportion of all neuro-WWLST deaths occurring in infants with SBI. Abbreviations: CUS, cranial ultrasound; cPVL, cystic periventricular leukomalacia; GA, gestational age; IVH, intraventricular hemorrhage; PVHI, periventricular hemorrhagic infarction; SBI, severe brain injury; neuro-WWLST, withdrawal, withholding, or non-escalation of life-sustaining treatment because of poor neurological prognosis.

Among 3081 eligible very preterm infants, 2841 had a complete recorded CUS1 assessment within 48 h after birth and constituted the CUS1-imaged denominator (Figure 1). Infants without complete CUS1 were more immature and had substantially higher early mortality, including deaths before NICU admission; they were therefore excluded from imaging-based analyses and are described separately in Supplementary Table S1. SBI was identified in 286/2841 infants (10.1%): PVHI in 117/286 (40.9%), the IVH3 entity in 94/286 (32.9%), and cPVL in 75/286 (26.2%). SBI prevalence declined from 22.2% at 22–24 weeks to 5.3% at ≥29 weeks. This decrease was mainly driven by PVHI and the IVH3 entity, whereas cPVL showed a less marked GA pattern (Supplementary Table S2). Across entities, median GA was lowest in PVHI and highest in cPVL (Table 1). Across the three study decades, overall SBI prevalence in the CUS1-imaged denominator declined from 12.5% in 1991–2000 to 8.7% in 2011–2020. Within SBI, the observed entity distribution shifted: PVHI accounted for 34.0% of SBI in 1991–2000 and 58.5% in 2011–2020, whereas the IVH3 entity and cPVL accounted for smaller proportions in the most recent decade. Median GA in the CUS1-imaged denominator remained stable at 29 weeks [IQR 27–31]. Deaths not classified as neuro-WWLST declined across epochs (11.0%, 12.8%, and 5.7%), survival increased to 92.5% in 2011–2020, and neuro-WWLST death rates within the CUS1-imaged denominator remained low and numerically similar across epochs (1.5%, 1.4%, and 1.8%) (Supplementary Tables S3–S7).

### 3.2. Entity-Specific Serial CUS Phenotypes and In-Hospital Outcomes

**Figure 2:**
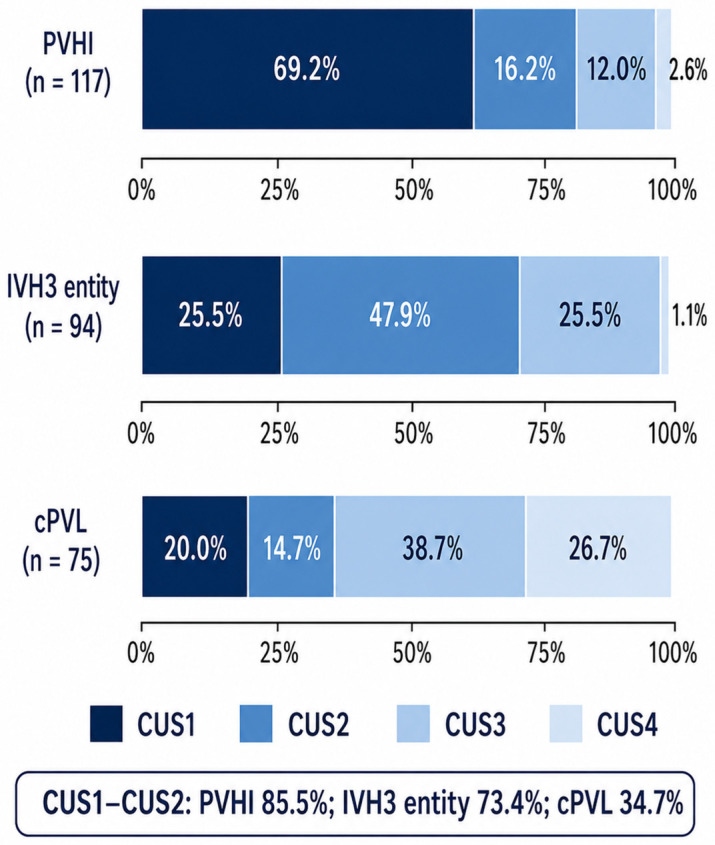
Timing of documented classifiability across serial CUS windows. Figure 2 shows the first prespecified CUS window in which each SBI entity met its operational classification criteria. PVHI debut was defined as the first CUS window showing the parenchymal lesion; IVH3 entity debut as the first CUS window reaching grade 3 IVH without meeting PVHI or cPVL criteria; and cPVL debut as the first CUS window meeting cPVL criteria. Darker shading indicates a higher proportion. Abbreviations: CUS, cranial ultrasound; cPVL, cystic periventricular leukomalacia; IVH, intraventricular hemorrhage; PVHI, periventricular hemorrhagic infarction.

PVHI was first documented as classifiable in CUS1–CUS2 in 100/117 cases (85.5%), with most cases already visible at CUS1. The IVH3 entity first reached grade 3 IVH in CUS1–CUS2 in 69/94 cases (73.4%), most often at CUS2. By contrast, cPVL more often became classifiable later, in CUS3–CUS4, in 49/75 cases (65.3%) (Figure 2). These timings refer to first documented classifiability within prespecified CUS windows, not to biological onset.

**Figure 3:**
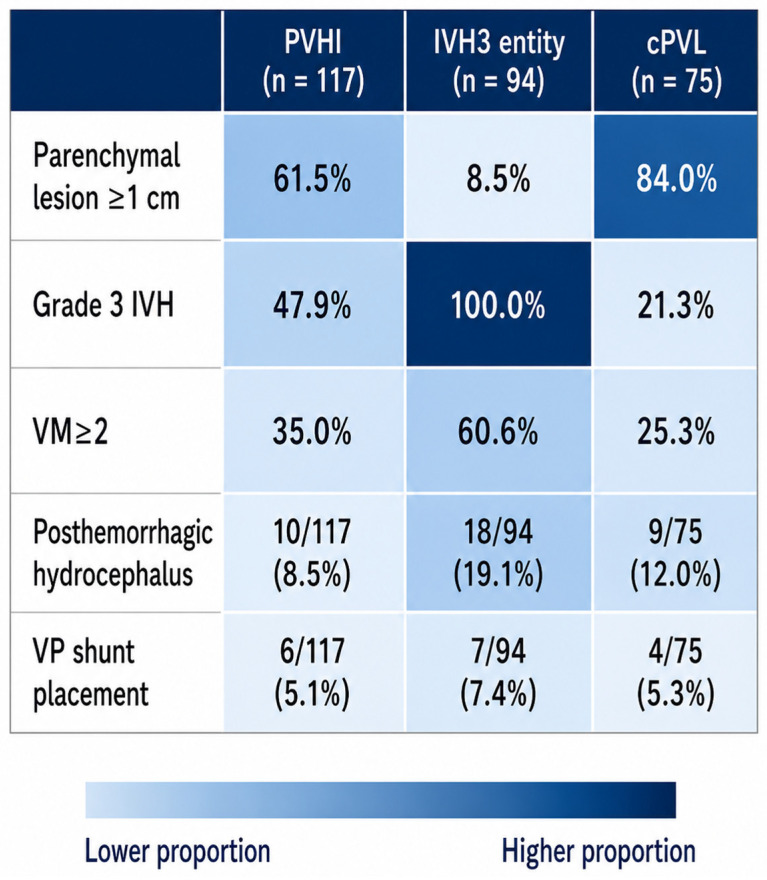
Maximum recorded imaging burden and ventricular clinical-course variables across SBI entities. Figure 3 summarizes the maximum recorded burden across the prespecified serial cranial ultrasound windows within each entity. Parenchymal lesion ≥ 1 cm, grade 3 IVH, and VM ≥ 2 refer to the maximum severity recorded during the serial ultrasound trajectory. Posthemorrhagic hydrocephalus and VP shunt placement were clinical-course variables and were not used to define imaging-domain burden. Darker shading indicates a higher proportion. Abbreviations: cPVL, cystic periventricular leukomalacia; IVH, intraventricular hemorrhage; PVHI, periventricular hemorrhagic infarction; VM, ventriculomegaly; VP, ventriculoperitoneal.

Parenchymal lesion ≥1 cm predominated in cPVL and PVHI but was uncommon in the IVH3 entity, whereas moderate-to-severe ventriculomegaly was most frequent in the IVH3 entity. Posthemorrhagic hydrocephalus was also most frequent in the IVH3 entity (18/94, 19.1%), while VP shunt placement was uncommon across entities (Figure 3). Posthemorrhagic hydrocephalus and VP shunt placement were treated as clinical-course variables, not as imaging severity domains.

Overall, 309/2841 infants (10.9%) died before discharge, including 45 neuro-WWLST deaths (1.6% of the CUS1-imaged denominator and 14.6% of all deaths) (Figure 1). The frequency of death following documented neuro-WWLST decreased from 6.1% at 22–24 weeks to 0.4% at ≥29 weeks (Supplementary Table S7). Of these, 43/45 (95.6%) occurred in infants with PVHI, the IVH3 entity, or cPVL (Figure 1). Within SBI, 89/286 infants (31.1%) died before discharge, and 43/89 (48.3%) of these deaths followed documented neuro-WWLST. By entity, neuro-WWLST accounted for 24/42 deaths (57.1%) in PVHI, 9/28 (32.1%) in the IVH3 entity, and 10/19 (52.6%) in cPVL (Table 1; Supplementary Table S8).

### 3.3. Outcome-Group Comparisons Using Common Maximal-Burden CUS

Table 2 summarizes the primary cross-outcome imaging comparison using common maximal-burden CUS. Compared with survivors, deaths following documented neuro-WWLST occurred in infants with lower GA and BW and showed an earlier, more multidomain maximal-burden profile. Maximal-burden CUS was selected in CUS1–CUS2 in 76.7% of deaths following documented neuro-WWLST versus 35.0% of survivors. At common maximal-burden CUS, deaths following documented neuro-WWLST more often showed parenchymal lesion ≥ 1 cm (81.4% vs. 45.7%), grade 3 IVH (72.1% vs. 49.2%), ≥2 severe domains (69.8% vs. 42.6%), and severe three-domain involvement (37.2% vs. 8.6%; unadjusted OR 6.27, *p* < 0.001). Unadjusted descriptive odds ratios and *p* values for the full set of comparisons are reported in Supplementary Table S9.

Compared with deaths not classified as neuro-WWLST, deaths following documented neuro-WWLST more often showed parenchymal lesion ≥ 1 cm, VM ≥ 2, ≥2 severe domains, and severe three-domain involvement, whereas CUS1–CUS2 selection of maximal-burden CUS was similar between both death groups (Table 2; Supplementary Table S9). These comparisons are descriptive and were not intended for prediction or causal inference.

Sensitivity analyses supported the scan-selection strategy. Later-window availability differed markedly by outcome group: complete CUS4 was available in 193/197 survivors, compared with 8/46 deaths not classified as neuro-WWLST and 6/43 deaths following documented neuro-WWLST, reflecting survival-dependent imaging opportunity. Among deaths following documented neuro-WWLST, last-window CUS contained the maximal documented burden in all 43 infants and showed the same severe-domain count and severe three-domain status as maximal-burden CUS in all cases. Conversely, among non-neuro-WWLST infants, using last available CUS would have underestimated maximal documented burden in 97/243 infants (39.9%), supporting maximal-burden CUS as the primary cross-outcome comparator (Supplementary Tables S10–S13). Additional restricted adjusted sensitivity models for death following documented neuro-WWLST within SBI produced similar results when GA was replaced by BW (Supplementary Table S14).

**Figure 4:**
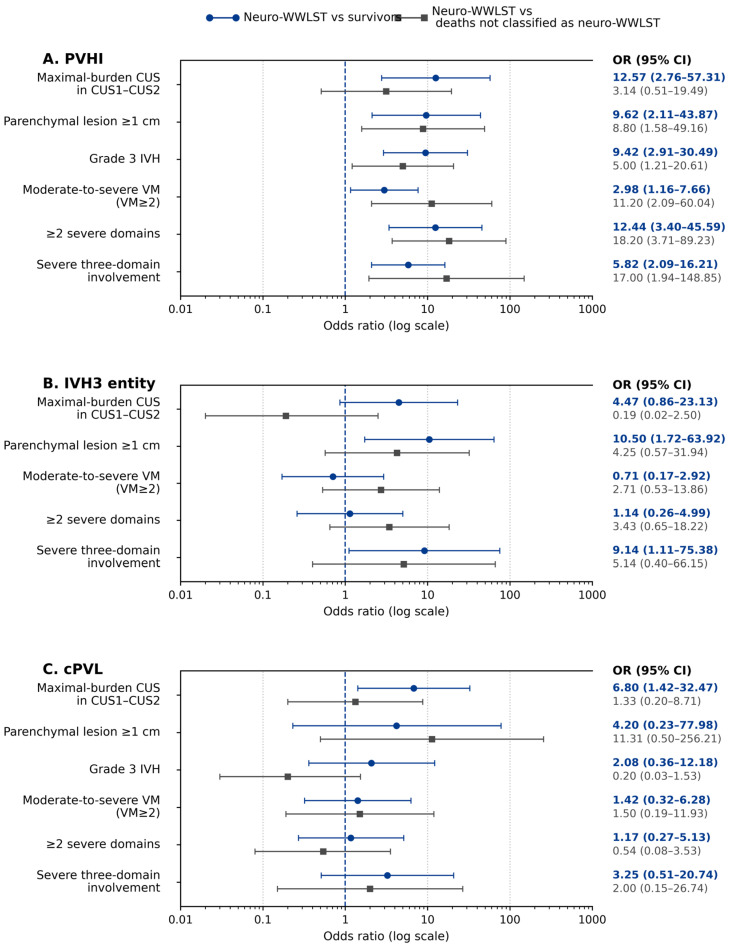
Entity-specific unadjusted descriptive odds ratios using common maximal-burden CUS. Figure 4 shows unadjusted descriptive odds ratios from the entity-specific common maximal-burden CUS comparisons reported in Supplementary Table S9. Odds ratios compare deaths following documented neuro-WWLST with survivors and with deaths not classified as neuro-WWLST within each SBI entity. Estimates are descriptive, unadjusted, and not intended for prediction or causal inference. Variables with invariant or non-estimable contrasts are omitted or labeled as not estimable. Abbreviations: CI, confidence interval; CUS, cranial ultrasound; IVH, intraventricular hemorrhage; OR, odds ratio; VM, ventriculomegaly; neuro-WWLST, withdrawal, withholding, or non-escalation of life-sustaining treatment because of poor neurological prognosis.

Entity-specific unadjusted descriptive ORs from the same common maximal-burden CUS comparison are shown in Figure 4 and detailed in Supplementary Table S9.

In PVHI, deaths following documented neuro-WWLST occurred in infants with lower GA and BW than survivors and showed a consistently higher hemorrhage-dominant maximal-burden profile, including more frequent CUS1–CUS2 maximal-burden selection, parenchymal lesion ≥ 1 cm, grade 3 IVH, VM ≥ 2, ≥2 severe domains, and severe three-domain involvement. Severe three-domain involvement was also more frequent in PVHI deaths following documented neuro-WWLST than in deaths not classified as neuro-WWLST (50.0% vs. 5.6%; unadjusted OR 17.00, *p* = 0.002). Consistent with this hemorrhage-dominant burden, death following documented neuro-WWLST was more frequent in PVHI with coexisting grade 3 IVH than in PVHI with maximal IVH < 3 (35.7% vs. 6.6%; unadjusted OR 7.92, 95% CI 2.50–25.05), including within the CUS1–CUS2 debut subgroup despite similar GA across strata (Supplementary Table S15).

In the IVH3 entity, deaths following documented neuro-WWLST also occurred in infants with lower GA and BW than survivors. At common maximal-burden CUS, parenchymal lesion ≥ 1 cm was more frequent among deaths following documented neuro-WWLST than among survivors (33.3% vs. 4.5%; unadjusted OR 10.50, *p* = 0.020). Severe three-domain involvement was uncommon but numerically higher among deaths following documented neuro-WWLST, with wide confidence intervals and non-significant Fisher testing, consistent with sparse subgroup data. Death following documented neuro-WWLST was more frequent in the IVH3 entity with additional parenchymal injury than in isolated IVH3 (50.0% vs. 5.8%; unadjusted OR 16.20, 95% CI 3.10–84.71) (Supplementary Table S16).

In cPVL, deaths following documented neuro-WWLST more often had maximal-burden CUS selected in CUS1–CUS2 than survivors (40.0% vs. 8.9%; unadjusted OR 6.80, *p* = 0.024), reflecting a small subset with early documented burden. However, within this entity-specific panel, grade 3 IVH, VM ≥ 2, ≥two severe domains, and severe three-domain involvement did not clearly distinguish deaths following documented neuro-WWLST from survivors (Figure 4; Supplementary Table S9). Neuro-WWLST proportions were similar in cPVL with and without coexisting grade 3 IVH (Supplementary Table S17).

### 3.4. Last-Window CUS Before Documented Neuro-WWLST Death

This analysis was restricted to infants whose deaths followed documented neuro-WWLST and describes the outcome-proximal imaging context. Severe involvement across all three imaging domains at the last-window CUS was most frequent in PVHI deaths (12/24, 50.0%), whereas posthemorrhagic hydrocephalus was most frequent in the IVH3 entity (4/9, 44.4%) and uncommon in PVHI (1/24, 4.2%) (Table 3). Posthemorrhagic hydrocephalus was considered a ventricular clinical-course variable, not an imaging severity domain. In an exploratory analysis restricted to neuro-WWLST deaths, early entity classifiability in CUS1–CUS2 was more frequent in the hemorrhage-dominant entities combined (PVHI or IVH3 entity, 32/33, 97.0%) than in cPVL (5/10, 50.0%; Fisher exact *p* = 0.001).

In PVHI and the IVH3 entity, the last-window CUS before death following documented neuro-WWLST usually occurred within the first week, whereas cPVL showed a more heterogeneous temporal profile, with last-window CUS more often extending into later surveillance windows (Table 4). In most cases, the last-window CUS coincided with the entity-debut window (29/43, 67.4%), supporting early outcome-proximal imaging classifiability in deaths following documented neuro-WWLST. The most evident interval change was observed in PVHI: severe involvement across all three domains increased from 6/24 (25.0%) at entity debut to 12/24 (50.0%) at last-window CUS (Table 3). Among PVHI deaths with delayed last-window CUS, this change was mainly driven by worsening ventriculomegaly, with additional worsening of IVH and parenchymal lesion severity in some cases (Table 4).

### 3.5. Neurodevelopmental Outcomes Among Survivors

**Figure 5:**
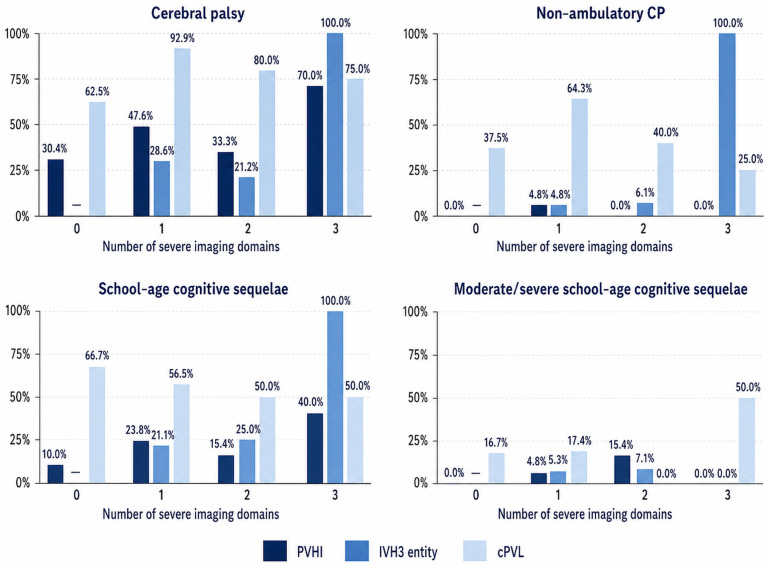
Neurodevelopmental outcomes among survivors by severe imaging-domain burden and severe brain injury entity. Figure 5 shows cerebral palsy, non-ambulatory cerebral palsy, clinically classified school-age cognitive sequelae, and moderate/severe clinically classified school-age cognitive sequelae among survivors, stratified by severe brain injury entity and number of severe imaging domains. Percentages are calculated among survivors with available follow-up data for each outcome. Severe imaging domains were parenchymal lesion ≥ 1 cm, grade 3 intraventricular hemorrhage, and ventriculomegaly grade ≥ 2, assessed at maximal-burden cranial ultrasound. Non-ambulatory CP was defined as Gross Motor Function Classification System levels III–V and was calculated among survivors with available CP follow-up. Denominators vary by entity, severe-domain category, and outcome; detailed n/N values and outcome categories are provided in Supplementary Tables S19–S21. A dash indicates that no survivors were present in that severe-domain category and therefore no percentage was calculated; 0.0% indicates an observed zero value among survivors with available follow-up data. Abbreviations: CP, cerebral palsy; cPVL, cystic periventricular leukomalacia; IVH, intraventricular hemorrhage; PVHI, periventricular hemorrhagic infarction.

Neurodevelopmental follow-up among survivors was available for CP at 2 years in 176/197 SBI survivors and for clinically classified school-age cognitive outcome in 155/197 (Supplementary Table S18). Overall, CP was present in 87/176 survivors (49.4%). Among survivors with CP and available GMFCS classification, 54/87 (62.1%) had ambulatory CP (GMFCS I–II) and 33/87 (37.9%) had non-ambulatory CP (GMFCS III–V) (Figure 5). Clinically classified school-age cognitive sequelae were present in 50/155 survivors (32.3%), including mild sequelae in 37/155 (23.9%) and moderate/severe sequelae in 13/155 (8.4%) (Supplementary Tables S19–S21).

Among survivors with severe involvement across all three imaging domains, CP was present in 13/17 (76.5%): 9/17 (52.9%) had ambulatory CP and 4/17 (23.5%) had non-ambulatory CP. Clinically classified school-age cognitive sequelae were present in 9/17 (52.9%): 7/17 (41.2%) had mild sequelae and 2/17 (11.8%) had moderate/severe sequelae (Figure 5; Supplementary Tables S19 and S21). Across SBI survivors, severe involvement across all three imaging domains was associated with a higher frequency of CP than involvement of fewer than three domains (13/17, 76.5% vs. 74/159, 46.5%; Fisher exact *p* = 0.022). This association remained after adjustment for GA, birth epoch, and SBI entity (adjusted OR 4.99, 95% CI 1.45–17.17; *p* = 0.011). Clinically classified school-age cognitive sequelae were also more frequent in the three-domain group (9/17, 52.9% vs. 41/138, 29.7%), although the unadjusted comparison did not reach conventional statistical significance (Fisher exact *p* = 0.096). In the adjusted model, severe three-domain involvement was associated with higher odds of cognitive sequelae (adjusted OR 3.72, 95% CI 1.19–11.65; *p* = 0.024). Sensitivity models replacing GA with BW showed similar directions and magnitudes of association (Supplementary Table S22). These analyses should be interpreted cautiously because the three-domain subgroup was small and cognitive outcomes were clinically classified from heterogeneous longitudinal sources rather than measured as harmonized continuous IQ endpoints. Additional methodological supporting analyses on scheduled CUS-window availability, complete three-domain availability, and maximal-burden CUS selection sensitivity are provided in Supplementary Tables S23–S26.

Entity-specific patterns suggested that CP frequency, functional severity, and motor phenotype differed across SBI entities. In PVHI, CP was present in 29/69 survivors with available CP assessment (42.0%). Almost all PVHI-associated CP was ambulatory (28/29, 96.6%), and all cases were unilateral/hemiparetic. Even among PVHI survivors with severe involvement across all three imaging domains, CP remained ambulatory and unilateral/hemiparetic, and cognitive sequelae, when present, were mild. In the IVH3 entity, CP was present in 16/57 survivors (28.1%); 10/16 (62.5%) were ambulatory, and the predominant motor phenotype was diplegic/paraparetic. The IVH3 subgroup with severe three-domain involvement was very small, limiting interpretation. In cPVL, CP was present in 42/50 survivors (84.0%) and was more often functionally severe: 26/42 CP cases (61.9%) were non-ambulatory. cPVL-associated CP was usually bilateral, most often diplegic/paraparetic or triparetic/tetraparetic. Clinically classified school-age cognitive sequelae were present in 23/41 cPVL survivors (56.1%). In cPVL, neither CP nor cognitive sequelae showed a clear stepwise gradient by number of severe imaging domains (Figure 5; Supplementary Tables S19–S21).

## 4. Discussion

In this single-center cohort of very preterm infants, SBI occurred in 286/2841 infants in the CUS1-imaged denominator (10.1%) and was concentrated among the most immature infants. Death following documented neuro-WWLST occurred in 45/2841 infants (1.6%), was also concentrated among the most immature infants and occurred almost exclusively among infants with the three prespecified SBI entities (43/45, 95.6%). Within SBI, death following documented neuro-WWLST accounted for almost half of in-hospital deaths (43/89, 48.3%). These findings are broadly consistent with contemporary very preterm cohorts showing that SBI remains an uncommon but high-impact complication concentrated among the most immature infants [1,6,7,8,34]. Direct comparison with previous studies is limited because many cohorts group severe hemorrhagic lesions under broad high-grade IVH categories, whereas our framework separates grade 3 IVH without PVHI from PVHI and cPVL as mutually exclusive SBI entities. In our cohort, the decline in overall SBI prevalence across decades was accompanied by an observed shift in entity distribution toward PVHI. This should be interpreted as a cohort-level reporting and classification pattern across a long study period, during which neonatal survival, imaging practice, ultrasound equipment, and reporting standards evolved [8,35,36].

The main contribution of this study is the separation of SBI into three mutually exclusive entities (PVHI, the IVH3 entity, and cPVL) and the use of routine serial CUS to describe lesion entity, timing of documented classifiability, maximal documented multidomain imaging burden, in-hospital outcome, and exploratory neurodevelopmental outcome among survivors. This framework allowed the primary outcome-group comparison to be interpreted using a common imaging summary, while last-window CUS provided a separate description of the outcome-proximal imaging context among deaths following documented neuro-WWLST. The framework was descriptive: it was intended to separate lesion label from the serial imaging trajectory in which the outcome occurred, not to use CUS as a decision rule, prognostic score, or causal determinant of outcome.

The timing patterns support the value of a time-aware approach to neonatal brain injury assessment. PVHI and the IVH3 entity were predominantly early documented CUS phenotypes, consistent with the known early timing of germinal matrix–IVH in preterm infants [15,37], whereas cPVL more often became classifiable later, in keeping with the delayed cystic evolution of white matter injury [9,23,26]. Previous serial CUS studies have described the temporal evolution of hemorrhagic injury, PVHI and cystic white matter injury [9,10,16,24,25]; our analysis extends this work by applying a common four-window, three-domain framework to mutually exclusive SBI entities and by explicitly separating survivors, deaths not classified as neuro-WWLST and deaths following documented neuro-WWLST. These timings indicate first documented classifiability within prespecified CUS windows, rather than biological onset. This distinction matters because SBI entities grouped under the same broad label may be documented at different stages of lesion evolution. An early hemorrhagic phenotype, a later cystic white matter injury phenotype, and the subsequent ventricular trajectory after hemorrhagic injury may all contribute to the clinical meaning of “severe brain injury”, while representing different points in lesion evolution and different clinical contexts.

The outcome analyses show why this distinction is clinically important. Major brain injury has been consistently linked with treatment-limitation pathways in extremely and very preterm infants [14,17,18,19,20,21,22,33], but previous studies have addressed the serial imaging context only partially. Sheehan et al. examined withdrawal of support after severe IVH and described CUS findings, including PVHI territorial burden, among infants who died, but did not compare serial multidomain CUS profiles across deaths following documented neuro-WWLST, survivors, and other deaths [22]. McCauley et al. showed regional variation in mortality after severe IVH and suggested that shared decision-making may be strongly influenced by ultrasound-based IVH assessment, but did not characterize the serial imaging phenotype of infants who died after treatment limitation [33]. James et al. reported marked intercentre variation in WWLST discussions among extremely preterm infants, but did not describe the corresponding CUS trajectory [20]. The present study adds this layer by using a common maximal-burden CUS definition for the primary cross-outcome comparison, while last-window CUS separately captured the outcome-proximal imaging context in deaths following documented neuro-WWLST.

Using the same maximal-burden CUS definition across outcome groups, deaths following documented neuro-WWLST showed greater multidomain imaging burden than survivors. The clearest summary marker was severe three-domain involvement, which was more frequent among deaths following documented neuro-WWLST than among survivors (37.2% vs. 8.6%). A similar multidomain pattern was observed when these deaths were compared with deaths not classified as neuro-WWLST, while early maximal-burden CUS selection was similar in both death groups. This suggests that the distinction was not simply a consequence of earlier imaging opportunity among infants who died, but reflected differences in maximal documented multidomain burden. These findings should nevertheless be interpreted as descriptive associations, not as evidence that CUS findings independently predicted or caused death following neuro-WWLST.

The neuro-WWLST outcome should be interpreted within its ascertainment context. In this study, neuro-WWLST referred only to deaths following documented withdrawal, withholding, or non-escalation of life-sustaining treatment because of poor neurological prognosis; the registry did not systematically capture counseling about poor neurological prognosis, treatment-limitation discussions, or decisions not followed by death. Decisions around treatment limitation are multifactorial and include GA, clinical course, comorbidities, expected survival, anticipated neurodevelopmental outcome, family values, and local practice. These decisions occurred within a single tertiary neonatal unit with long-standing continuity of care and progressive incorporation of family-centered developmental care, but practices, documentation, counseling, and thresholds for treatment limitation may still have evolved across epochs. Therefore, severe ultrasound findings should be understood as part of the documented clinical context in which these deaths occurred, not as a decision rule or as a complete representation of decision-making.

The last-window analysis provided an outcome-proximal description that would be missed by considering entity debut or maximal-burden summaries alone. This was most evident in PVHI. Severe three-domain involvement in PVHI deaths following documented neuro-WWLST increased from 6/24 (25.0%) at entity debut to 12/24 (50.0%) at last-window CUS. Among delayed PVHI deaths, severe three-domain involvement was absent when PVHI first became classifiable but present by the last-window CUS in 6/9 infants, mainly through worsening VM and IVH severity. This supports describing PVHI not only by lesion label or debut findings, but by its evolving parenchymal, hemorrhagic, and ventricular burden. This interpretation is consistent with previous PVHI and hemorrhagic-injury literature showing that outcome depends on lesion extent, topography, associated IVH, VM, and posthemorrhagic hydrocephalus rather than on the lesion label alone [10,11,12,13,14,29], and with structured CUS approaches emphasizing detailed hemorrhagic, parenchymal, and ventricular reporting [16,25].

In the IVH3 entity and cPVL, the pattern was different. In the IVH3 entity, grade 3 IVH itself could not discriminate outcome context because it defined the entity; the clinically relevant distinction was whether grade 3 IVH was accompanied by additional parenchymal or ventricular burden. Deaths following documented neuro-WWLST showed more additional parenchymal and multidomain burden than survivors, although estimates were sparse and uncertain. These deaths were also more frequent in the IVH3 entity with additional parenchymal injury than in isolated IVH3, supporting interpretation of grade 3 IVH as part of a broader injury profile rather than as an isolated scan-level label [2,16,30]. In cPVL, by contrast, deaths following documented neuro-WWLST were not clearly distinguished from survivors by additional hemorrhagic or ventricular burden. The main distinction was temporal: these deaths more often had maximal-burden CUS selected in CUS1–CUS2, reflecting a subset with early documented burden. This should not be interpreted as evidence that early cPVL independently increases the probability of treatment limitation or death; rather, it shows that the clinical meaning of timing differs by entity. In PVHI, outcome-proximal imaging may capture interval accumulation of multidomain burden, whereas in cPVL it may capture early recognition of a severe white matter injury phenotype.

The survivor outcome findings should be interpreted as exploratory, but they reinforce the entity-specific nature of the imaging framework. Among SBI survivors with available follow-up, CP was present in 87/176 (49.4%) and clinically classified school-age cognitive sequelae in 50/155 (32.3%). Severe three-domain involvement identified a higher-risk subset, with more frequent CP (76.5% vs. 46.5%) and clinically classified cognitive sequelae (52.9% vs. 29.7%) than in survivors with fewer than three severe domains. However, domain count did not translate into a uniform or simply stepwise outcome pattern, reflecting entity mix, small strata, and different lesion mechanisms.

The motor-phenotype data further support entity-specific interpretation. In PVHI, severe three-domain involvement identified a subgroup with more frequent CP, but PVHI-associated CP was almost always ambulatory (28/29) and uniformly unilateral/hemiparetic. In cPVL, CP was more frequent overall (42/50) and more often non-ambulatory (26/42) and bilateral, consistent with the motor outcome relevance of cystic white matter injury [32,38]. Nevertheless, the outcome was not uniform: CP was absent in 8/50 (16.0%) cPVL survivors with available CP data, consistent with reports that a subset of infants with cPVL may have good neurological outcome [39]. In the IVH3 entity, motor phenotype was more heterogeneous and the severe three-domain subgroup was too small to support robust entity-specific inference. Overall, these findings support structured, entity-specific interpretation of serial CUS: severe multidomain burden indicates increased risk, but it does not define a uniform functional outcome and should not be used as a deterministic prognostic label. Cognitive findings require particular caution because school-age cognitive sequelae were clinically classified from heterogeneous longitudinal information rather than measured as harmonized continuous IQ endpoints.

These findings have practical implications for neonatal imaging, communication about prognosis, and cohort comparison. In clinically unstable very preterm infants, serial CUS may be the only neuroimaging record available during the acute NICU course, because MRI is often performed later and may not be feasible before early deterioration or death [26,27,28]. Its value therefore lies not only in detecting major lesions, but also in documenting their timing and evolution during a period when clinical trajectory may still be changing. These data may help frame clinical communication more transparently: severe multidomain imaging burden indicates increased risk, but the expected functional range remains entity-specific and should be interpreted alongside lesion location, clinical course, comorbidities, brain maturation, and broader neonatal trajectory [15,16]. This aligns with recommendations for appropriately timed, structured, and descriptively reported neonatal CUS examinations [23,24], and may support cohort comparison by separating lesion entity, documented imaging burden, timing, survival-dependent imaging opportunity, and treatment-limitation pathways in long-term cohort data.

The main strengths of this study are the large prospectively maintained tertiary NICU cohort, prespecified serial CUS windows, separation of SBI into three mutually exclusive entities, and a three-domain CUS framework covering parenchymal lesion, IVH and ventriculomegaly. The primary cross-outcome comparison used a common maximal-burden CUS definition across all outcome groups, while last-window CUS provided an outcome-proximal description among deaths following documented neuro-WWLST. The study also reports the CUS1-imaged denominator, CUS-window availability, scan-selection sensitivity analyses, and survivor outcomes including CP, GMFCS functional severity, motor phenotype and clinically classified school-age cognitive sequelae.

Several limitations should be acknowledged. First, this was a retrospective single-center study spanning 30 years. Neonatal care, survival, family-centered developmental-care practices, counseling about poor neurological prognosis, ultrasound equipment, image quality, terminology and reporting standards may have changed across epochs. Birth epoch was described and included in selected adjusted analyses, but residual secular effects cannot be excluded. The observed shift in entity distribution toward PVHI should therefore be interpreted as a cohort-level reporting and classification pattern, not as definitive evidence of changing lesion epidemiology. Classification relied on contemporaneous routine radiology reports; images were not systematically re-reviewed, interobserver agreement was not assessed, and MRI could not serve as a uniform adjudication standard because availability was epoch-, survival- and outcome-dependent.

Second, imaging analyses were restricted to infants with complete recorded CUS1, so SBI prevalence, mortality and neuro-WWLST estimates apply to the CUS1-imaged denominator rather than to the full eligible very preterm population. Later CUS availability was also survival- and clinical-course dependent. CUS-window availability and scan-selection sensitivity analyses addressed this issue, but timing findings still represent documented classifiability within available prespecified windows, not biological lesion onset. Neuro-WWLST ascertainment was limited to deaths following documented withdrawal, withholding, or non-escalation of life-sustaining treatment because of poor neurological prognosis; non-fatal discussions or decisions were not systematically captured.

Finally, entity-specific neuro-WWLST and survivor outcome subgroups were small, no multiplicity adjustment was applied, and adjusted models were intentionally restricted. BW was examined in sensitivity analyses replacing GA, and that the two variables were not entered together in the same model because of collinearity. Other neonatal morbidities and treatment exposures were not included to avoid overfitting and because their ascertainment and clinical meaning may have varied across the study period. School-age cognitive sequelae were clinically classified from heterogeneous longitudinal information rather than measured as a harmonized continuous IQ endpoint. These data should therefore be interpreted as descriptive associations rather than prediction or causal inference.

Because treatment-limitation practices and neurodevelopmental follow-up pathways vary across units and countries, the imaging and outcome context of neuro-WWLST cannot be assumed to be uniform. Multicenter studies using standardized postnatal CUS windows, consistent three-domain definitions, mutually exclusive SBI entities, explicit ascertainment of treatment-limitation discussions and decisions, time-comparable MRI where feasible, and harmonized CP, GMFCS, motor-phenotype and cognitive-outcome reporting would help determine whether the patterns observed here are reproducible across different care settings [19,20,33,40,41]. This would provide a more transparent basis for distinguishing lesion biology, documented imaging burden, survival-dependent imaging opportunity, and treatment-limitation practice when comparing SBI-related mortality and survivor outcomes.

## 5. Conclusions

In this single-center cohort of very preterm infants, SBI was concentrated among the most immature infants and was present in most deaths following documented neuro-WWLST. Serial CUS showed that PVHI, the IVH3 entity, and cPVL had distinct time-dependent phenotypes with different patterns of parenchymal, hemorrhagic, and ventricular involvement. Among survivors, severe multidomain involvement identified a higher-risk subgroup for CP and clinically classified school-age cognitive sequelae, but outcomes differed by SBI entity, motor phenotype, and functional severity, and were not deterministic. These findings support a structured, descriptive CUS approach that separates lesion entity, timing of documented classifiability, and multidomain burden, and may help improve the transparency of cohort-level descriptions of SBI trajectories, deaths following documented neuro-WWLST, and survivor outcomes.

## Figures and Tables

**Table 1:** Clinical profile and in-hospital outcomes across severe brain injury entities.

Descriptor	PVHI (*n* = 117)	IVH3 Entity (*n* = 94)	cPVL (*n* = 75)
**Perinatal descriptors**			
Gestational age, weeks, median (IQR)	26 (25–29)	27 (25–28)	28 (26–30)
Birth weight, g, median (IQR)	900 (700–1080)	1030 (785–1199)	1000 (823–1244)
**Birth epoch**			
1991–2000	32/117 (27.4%)	30/94 (31.9%)	32/75 (42.7%)
2001–2010	23/117 (19.7%)	42/94 (44.7%)	21/75 (28.0%)
2011–2020	62/117 (53.0%)	22/94 (23.4%)	22/75 (29.3%)
**In-hospital outcomes**			
Survived to discharge	75/117 (64.1%)	66/94 (70.2%)	56/75 (74.7%)
All in-hospital deaths	42/117 (35.9%)	28/94 (29.8%)	19/75 (25.3%)
Death without neuro-WWLST	18/117 (15.4%)	19/94 (20.2%)	9/75 (12.0%)
Neuro-WWLST death	24/117 (20.5%)	9/94 (9.6%)	10/75 (13.3%)
Neuro-WWLST among deaths	24/42 (57.1%)	9/28 (32.1%)	10/19 (52.6%)

Values are *n* (%) unless otherwise stated. Bold text indicates section headings within the table. SBI entities were mutually exclusive. PVHI was classified irrespective of coexisting IVH grade. The IVH3 entity comprised infants reaching grade 3 IVH without meeting PVHI or cPVL criteria. Birth-epoch percentages are calculated within each SBI entity. Death not classified as neuro-WWLST and death following documented neuro-WWLST sum to all in-hospital deaths. Neuro-WWLST among deaths was calculated among infants who died before discharge within each SBI entity. Abbreviations: cPVL, cystic periventricular leukomalacia; IVH, intraventricular hemorrhage; IVH3, grade 3 intraventricular hemorrhage; PVHI, periventricular hemorrhagic infarction; SBI, severe brain injury; neuro-WWLST, withdrawal, withholding, or non-escalation of life-sustaining treatment because of poor neurological prognosis.

**Table 2:** Outcome-group comparisons using common maximal-burden CUS in infants with severe brain injury.

Variable	Survivors (*n* = 197)	Death Without Neuro-WWLST (*n* = 46)	Neuro-WWLST Death (*n* = 43)
**A. Clinical case-mix descriptors**			
Gestational age, weeks, median (IQR)	28 (26–29)	26 (25–29)	26 (24.5–26.5)
Birth weight, g, median (IQR)	1010 (830–1230)	855 (675–1084.8)	775 (663–985)
**B. Timing of selected maximal-burden CUS**			
Maximal-burden CUS in CUS1–CUS2	69/197 (35.0%)	35/46 (76.1%)	33/43 (76.7%)
**C. Maximal-burden CUS imaging features**			
Parenchymal lesion ≥ 1 cm (LP4)	90/197 (45.7%)	18/46 (39.1%)	35/43 (81.4%)
Grade 3 IVH	97/197 (49.2%)	33/46 (71.7%)	31/43 (72.1%)
VM ≥ 2	79/197 (40.1%)	10/46 (21.7%)	22/43 (51.2%)
≥Two severe domains	84/197 (42.6%)	16/46 (34.8%)	30/43 (69.8%)
Severe three-domain involvement	17/197 (8.6%)	3/46 (6.5%)	16/43 (37.2%)
**D. Ventricular clinical-course variables**			
Posthemorrhagic hydrocephalus	30/197 (15.2%)	0/46 (0.0%)	7/43 (16.3%)
VP shunt placement	17/197 (8.6%)	0/46 (0.0%)	0/43 (0.0%)

Values are *n* (%) unless otherwise stated. Bold text indicates section headings within the table. Maximal-burden CUS was selected using the same prespecified hierarchical rule in all outcome groups. Severe imaging domains were parenchymal lesion ≥ 1 cm, grade 3 IVH, and VM ≥ 2. Severe three-domain involvement denotes concurrent presence of all three severe imaging domains at maximal-burden CUS. Posthemorrhagic hydrocephalus and VP shunt placement were clinical-course variables and were not used to define the severe-domain count. Detailed outcome-group comparisons, including entity-specific panels and unadjusted descriptive odds ratios, are reported in Supplementary Table S9. Abbreviations: CUS, cranial ultrasound; IQR, interquartile range; IVH, intraventricular hemorrhage; VM, ventriculomegaly; VP, ventriculoperitoneal; neuro-WWLST, withdrawal, withholding, or non-escalation of life-sustaining treatment because of poor neurological prognosis.

**Table 3:** Entity-debut and last-window CUS imaging burden among neuro-WWLST deaths.

Descriptor	PVHI (*n* = 24)	IVH3 Entity (*n* = 9)	cPVL (*n* = 10)
**A. Imaging burden at entity-debut CUS**			
Parenchymal lesion ≥ 1 cm	19/24 (79.2%)	2/9 (22.2%)	9/10 (90.0%)
Grade 3 IVH	15/24 (62.5%)	9/9 (100.0%)	2/10 (20.0%)
VM ≥ 2	7/24 (29.2%)	3/9 (33.3%)	2/10 (20.0%)
≥Two severe imaging domains	14/24 (58.3%)	4/9 (44.4%)	2/10 (20.0%)
Severe three-domain involvement	6/24 (25.0%)	1/9 (11.1%)	1/10 (10.0%)
**B. Imaging burden at last-window CUS before neuro-WWLST death**			
Parenchymal lesion ≥ 1 cm	22/24 (91.7%)	3/9 (33.3%)	10/10 (100.0%)
Grade 3 IVH	20/24 (83.3%)	9/9 (100.0%)	2/10 (20.0%)
VM ≥ 2	14/24 (58.3%)	5/9 (55.6%)	3/10 (30.0%)
≥Two severe imaging domains	21/24 (87.5%)	6/9 (66.7%)	3/10 (30.0%)
Severe three-domain involvement	12/24 (50.0%)	2/9 (22.2%)	2/10 (20.0%)
**C. Ventricular clinical-course variable documented up to the last-window CUS**			
Posthemorrhagic hydrocephalus	1/24 (4.2%)	4/9 (44.4%)	2/10 (20.0%)

Values are *n* (%). Bold text indicates section headings within the table. This table is restricted to infants whose deaths followed documented neuro-WWLST and describes outcome-proximal imaging context. Entity-debut CUS was the first CUS window meeting criteria for the corresponding SBI entity. Last-window CUS was the last recorded CUS within the prespecified windows before death. Severe imaging domains were parenchymal lesion ≥ 1 cm, grade 3 IVH, and VM ≥ 2. Severe three-domain involvement denotes concurrent presence of all three severe imaging domains. Posthemorrhagic hydrocephalus was a clinical-course variable and was not used to define the severe-domain count. Abbreviations: CUS, cranial ultrasound; cPVL, cystic periventricular leukomalacia; IVH, intraventricular hemorrhage; IVH3, grade 3 intraventricular hemorrhage; PVHI, periventricular hemorrhagic infarction; SBI, severe brain injury; VM, ventriculomegaly; neuro-WWLST, withdrawal, withholding, or non-escalation of life-sustaining treatment because of poor neurological prognosis.

**Table 4:** Outcome-proximal CUS timing and interval change among neuro-WWLST deaths.

Descriptor	PVHI (*n* = 24)	IVH3 Entity (*n* = 9)	cPVL (*n* = 10)
**A. Last-window CUS timing**			
Last-window CUS in CUS1–CUS2	21/24 (87.5%)	7/9 (77.8%)	4/10 (40.0%)
Last-window CUS in CUS3–CUS4	3/24 (12.5%)	2/9 (22.2%)	6/10 (60.0%)
**B. Window delay from entity debut to last-window CUS**			
0 windows	15/24 (62.5%)	7/9 (77.8%)	7/10 (70.0%)
1 window	7/24 (29.2%)	0/9 (0.0%)	3/10 (30.0%)
≥2 windows	2/24 (8.3%)	2/9 (22.2%)	0/10 (0.0%)
**C. Gain in severe imaging domains among delayed cases**			
Delayed timing denominator	9	2	3
+0 domains	0/9 (0.0%)	0/2 (0.0%)	1/3 (33.3%)
+1 domain	5/9 (55.6%)	1/2 (50.0%)	2/3 (66.7%)
+≥2 domains	4/9 (44.4%)	1/2 (50.0%)	0/3 (0.0%)
**D. Domain worsening among delayed cases**			
Parenchymal lesion worsening	3/9 (33.3%)	1/2 (50.0%)	1/3 (33.3%)
IVH worsening	5/9 (55.6%)	0/2 (0.0%)	0/3 (0.0%)
Ventriculomegaly worsening	8/9 (88.9%)	2/2 (100.0%)	2/3 (66.7%)

Values are *n* (%) unless otherwise stated. Bold text indicates section headings within the table. This table is restricted to infants whose deaths followed documented neuro-WWLST. Last-window CUS was the last recorded CUS within the prespecified windows before death. Window delay refers to the number of scheduled CUS windows between entity debut and last-window CUS; 0 indicates the same window. Sections C and D are restricted to delayed cases, defined as last-window CUS occurring at least one window after entity debut. Severe imaging domains were parenchymal lesion ≥ 1 cm, grade 3 IVH, and VM ≥ 2. Gain in severe domains denotes the increase in the number of severe imaging domains from entity debut to last-window CUS. Domain worsening denotes interval worsening in the corresponding domain grade between entity debut and last-window CUS. Abbreviations: CUS, cranial ultrasound; cPVL, cystic periventricular leukomalacia; IVH, intraventricular hemorrhage; IVH3, grade 3 intraventricular hemorrhage; PVHI, periventricular hemorrhagic infarction; VM, ventriculomegaly; neuro-WWLST, withdrawal, withholding, or non-escalation of life-sustaining treatment because of poor neurological prognosis.

## Data Availability

The data presented in this study are available on reasonable request from the corresponding author. The data are not publicly available because they derive from a clinical registry of very preterm infants and contain sensitive health information subject to institutional, ethical, and privacy restrictions.

## Supplementary Materials

The following supporting information can be downloaded at https://www.mdpi.com/article/10.3390/children13070844/s1: Supplementary Methods S1: Cohort assembly and CUS1-imaged denominator. Supplementary Methods S2: CUS surveillance windows, MRI availability, and available registry data. Supplementary Methods S3: Operational CUS grading details. Supplementary Methods S4: SBI entity definitions, mutual exclusivity, laterality, and extent. Supplementary Methods S5: In-hospital outcomes, unit context and neuro-WWLST ascertainment. Supplementary Methods S6: Scan-selection rules, robustness and sensitivity analyses, statistical analysis, and missing data. Supplementary Methods S7: Neurodevelopmental follow-up and survivor outcome analyses. Supplementary Table S1: Eligible infants with and without complete CUS1. Supplementary Table S2: Severe brain injury prevalence by gestational age group in the CUS1-imaged cohort. Supplementary Table S3: SBI prevalence in the CUS1-imaged denominator, by decade. Supplementary Table S4: Distribution of SBI entities within SBI, by decade. Supplementary Table S5: Cohort outcomes by decade in the CUS1-imaged denominator. Supplementary Table S6: Cohort outcomes by gestational age group within each decade. Supplementary Table S7: Cohort outcomes by gestational age group in the CUS1-imaged denominator. Supplementary Table S8: SBI entities, outcome distribution, and within-entity subgroups defined by coexisting grade 3 IVH or additional parenchymal injury. Supplementary Table S9: Outcome-group comparisons using common maximal-burden CUS. Supplementary Table S10: Scheduled complete CUS-window availability by outcome group and SBI entity. Supplementary Table S11: Concordance between last-window CUS and maximal-burden CUS among neuro-WWLST deaths. Supplementary Table S12: Last available CUS versus maximal-burden CUS among survivors and deaths not classified as neuro-WWLST. Supplementary Table S13: Fixed early-window CUS1–CUS2 imaging burden. Supplementary Table S14: Restricted adjusted and sparse-data sensitivity models. Supplementary Table S15: PVHI: gestational age and neuro-WWLST by coexisting grade 3 IVH, overall and by debut window. Supplementary Table S16: IVH3 entity: gestational age and neuro-WWLST by additional parenchymal injury versus isolated IVH3. Supplementary Table S17: cPVL: gestational age and neuro-WWLST by coexisting grade 3 IVH, overall and by debut window. Supplementary Table S18: Neurodevelopmental follow-up availability among SBI survivors. Supplementary Table S19: Cerebral palsy and GMFCS among survivors by SBI entity and severe-domain count. Supplementary Table S20: Cerebral palsy motor phenotype among survivors with CP by SBI entity and severe-domain count. Supplementary Table S21: Clinically classified school-age cognitive sequelae among survivors by SBI entity and severe-domain count. Supplementary Table S22: Exploratory adjusted associations between severe three-domain involvement and survivor outcomes. Supplementary Table S23: Scheduled CUS window availability across SBI entities. Supplementary Table S24: Complete three-domain availability across SBI entities. Supplementary Table S25: Algorithm-resolution profile for maximal-burden CUS selection in PVHI without neuro-WWLST. Supplementary Table S26: Sensitivity to alternative tie-breaking rules for maximal-burden CUS selection in PVHI without neuro-WWLST. Supplementary Table S27: STROBE checklist for cohort studies.
