# Supplementary material for: Three-Domain Serial Cranial Ultrasound Phenotypes and Outcomes in Very Preterm Infants with Severe Brain Injury: A Single-Center Cohort Study
Source: Children (Basel). 2026 Jun 23;13(7):844. doi: 10.3390/children13070844 (PMC13406346; doi:10.3390/children13070844)
Supplement: Supplementary file 1 [file children-13-00844-s001.zip › children-4353582-supplementary.pdf]

SUPPLEMENTARY METHODS AND TABLES

Three-Domain Serial Cranial Ultrasound Phenotypes and Outcomes in Very Preterm Infants with Severe Brain Injury: A Single-Center Cohort Study

SUPPLEMENTARY METHODS (S1–S7)

SUPPLEMENTARY TABLES (S1–S26)

Supplementary Table S27. STROBE checklist for cohort studies

Supplementary Methods S1. Cohort assembly and CUS1-imaged denominator.

▪ **Cohort assembly.** Eligible infants were live-born very preterm infants (<32 weeks' gestational age [GA] and/or birth weight [BW] ≤1,500 g) delivered at the study center and/or admitted within the first 48 hours after birth between 1991 and 2020 (n=3,081). No formal sample size calculation was performed; all eligible infants recorded in the prospectively maintained registry during the study period were considered.

▪ **CUS1-imaged denominator.** The imaging-based analytic denominator (CUS1-imaged denominator) was defined as infants with a complete recorded CUS1 assessment, defined by availability of all three prespecified imaging domains within 48 hours after birth (n=2,841) (Figure 1). Infants without a complete recorded CUS1 assessment were excluded from imaging-based analyses (n=240), including deaths before NICU admission (n=113) and infants admitted to the NICU without a complete recorded CUS1 assessment because of early death, transfer, or other logistical reasons (n=127). In-hospital outcomes were assessed until discharge or death. Neurodevelopmental outcomes among survivors were assessed through the longitudinal follow-up program, including CP assessment at approximately 2 years' corrected age and cognitive outcome classification up to school age when available. The CUS1-imaged analytic denominator and comparison with eligible infants without complete CUS1 are reported in Supplementary Table S1.

Supplementary Methods S2. CUS surveillance windows, MRI availability, and available registry data.

▪ **CUS surveillance windows and acquisition context.** Routine CUS surveillance was summarized using four prespecified windows: CUS1 (≤48 hours), CUS2 (around day 7), CUS3 (around day 28), and CUS4 (term-equivalent age [TEA] or discharge, whichever occurred first). CUS examinations were performed for clinical care and documented prospectively in contemporaneous radiology reports by the neonatal radiology team, including senior pediatric radiologists. Interobserver reliability was not formally assessed. For this study, grades were extracted from the contemporaneous clinical reports; images were not systematically reclassified retrospectively. For analysis, each window was represented by a single prespecified surveillance assessment. If more than one CUS occurred within a window, the protocol-designated surveillance study was used when explicitly identifiable; otherwise, the examination closest to the target day for that window was selected. Parenchymal lesion pattern (LP), intraventricular hemorrhage (IVH), and ventriculomegaly (VM) grades from the selected within-window assessment were used in all analyses. Scheduled complete CUS-window availability by outcome group and SBI entity is reported in Supplementary Table S10.

▪ **MRI availability across epochs.** MRI was available in a subset of infants but was not incorporated into the main analytical framework because availability was not uniform across epochs or outcome groups. In infants surviving to term-equivalent age, MRI became more routinely performed from the 2000s onward, whereas in the 1990s MRI was more often obtained later during neurologic follow-up when neurodevelopmental abnormalities or cerebral palsy were suspected. This temporal and outcome-dependent availability limited its use for time-comparable cohort-wide analysis. MRI availability was also survival-dependent and outcome-dependent, because many infants who died early, including infants whose deaths followed documented neuro-WWLST, did not survive long enough to undergo term-equivalent MRI. Therefore, MRI could not be used as a time-comparable or outcome-comparable adjudication standard across the full cohort. Lesion classification therefore relied on serial CUS as recorded in routine clinical practice; subtle hemorrhagic findings may have been underdetected in earlier epochs, and MRI could not serve as a cohort-wide adjudication standard.

▪ **Additional clinical variables in the registry.** The registry also contains a broader set of clinical variables recorded during NICU hospitalization, including non-neurologic complications and aspects of the overall clinical course. These variables were not included in the present analysis, which was

intentionally focused on serial CUS patterns together with GA, BW, and birth epoch. Restricted adjusted and sparse-data sensitivity models were fitted only where estimable, as described in Supplementary Methods S6 and Supplementary Table S14.

Supplementary Methods S3. Operational CUS grading details.

▪ **Parenchymal injury.** Parenchymal lesion pattern (LP) grades were abstracted from the routine clinical report for each CUS window. Unless otherwise specified, LP refers to the grade recorded at that window, and peak LP denotes the maximum LP grade recorded across follow-up. LP was coded on an ordinal 0–4 scale reflecting parenchymal involvement: LP0, no parenchymal lesion; LP1–LP2, focal non-cystic periventricular echogenic lesions <1 cm adjacent to the ventricular wall, with echogenicity equal to the choroid plexus (LP1) or greater than the choroid plexus (LP2); LP3, cystic/porencephalic lesion <1 cm; and LP4, hyperechogenic or cystic lesion ≥1 cm and/or extending ≥1 cm from the ventricular wall. In the main analyses, parenchymal injury was simplified to parenchymal lesion ≥1 cm.

▪ **IVH.** IVH was coded on an ordinal scale (IVH0–IVH3) using a Volpe-based approach as reflected in routine reporting. When laterality differed, the higher-grade side was used for analysis. In the main text, grade 3 IVH refers to this scan-level severity descriptor, irrespective of whether it was present initially or reached later by progression.

▪ **Ventriculomegaly.** VM grading used measurement-based thresholds recorded in routine reporting, based on thalamo-occipital distance (TOD) and atrial (trigonal) diameter, measured inner-to-inner on standard clinical acquisition planes. Prespecified bands were VM0 (none), TOD <20 mm and atrial diameter <10 mm; VM1 (mild), TOD 20–24.9 mm and/or atrial diameter 10–12 mm; VM2 (moderate), TOD 25–29.9 mm and/or atrial diameter 13–15 mm; and VM3 (severe), TOD ≥30 mm and/or atrial diameter >15 mm. If TOD and atrial diameter fell into different bands, the higher VM grade was assigned. In the main analyses, clinically relevant ventriculomegaly was defined as VM≥2. Hydrocephalus was captured as a documented clinical-course variable from routine radiology or clinical records and was not used as an independent severity-defining domain.

Supplementary Methods S4. SBI entity definitions, mutual exclusivity, laterality, and extent.

▪ The three SBI entities were defined a priori from serial CUS and treated as mutually exclusive.

• **PVHI.** PVHI was defined as a periventricular hemorrhagic parenchymal lesion adjacent to the lateral ventricle, with hemorrhagic/venous-type morphology and serial persistence and/or cystic/porencephalic evolution. Lesions could be separate from or partially connected to the ventricle and were classified irrespective of associated IVH grade. Case attribution relied on lesion topography, morphology, and serial evolution across prespecified protocol windows rather than on IVH status alone. Once the serial pattern was adjudicated as PVHI, operational debut was assigned to the first CUS window showing the parenchymal lesion; operational debut for PVHI was therefore the first CUS window with LP>0.

• **IVH3 entity.** The IVH3 entity comprised infants reaching grade 3 IVH during follow-up without meeting PVHI or cPVL criteria, including isolated grade 3 IVH and grade 3 IVH with additional parenchymal injury. Operational debut was the first CUS window with grade 3 IVH. Additional parenchymal injury included arterial infarction/hemorrhage, either lobar or cerebellar, and two mixed focal parenchymal lesions with cystic and hemorrhagic features that did not fulfill PVHI or cPVL criteria. These cases were assigned to the IVH3 entity under the prespecified mutual-exclusivity rule.

• **cPVL.** cPVL was defined as periventricular cysts compatible with cystic periventricular leukomalacia; isolated echodensities or prolonged flares without cyst formation were excluded. In distinguishing cPVL from PVHI, particular weight was given to lesion topography, non-hemorrhagic versus hemorrhagic morphology, and serial evolution. Operational debut for cPVL was the first CUS window meeting cPVL criteria, corresponding operationally to LP≥3 with compatible cystic white matter injury morphology.

▪ **Laterality and extent.** Laterality (unilateral/bilateral) and lobar distribution or extent were abstracted from routine reports when available. Multilobar involvement was defined as involvement of ≥2 lobes when explicitly described. Missingness reflects absent documentation in the clinical report. For severity summarization, prespecified high-burden findings were parenchymal lesion ≥1 cm (corresponding to LP4), grade 3 IVH, and VM≥2. Severe involvement across all three domains was defined as the concurrent presence of these three findings. These operational definitions were intended to support reproducible serial CUS phenotyping within the cohort and mutual exclusivity across SBI entities; they

were not intended as MRI-based adjudication of underlying mechanism or as a neuro-WWLST decision rule.

Supplementary Methods S5. In-hospital outcomes, unit context and neuro-WWLST ascertainment.

▪ **In-hospital outcomes.** In-hospital outcomes were categorized as survival to discharge, death not classified as neuro-WWLST in the registry, and neuro-WWLST death. Neuro-WWLST death was defined as in-hospital death following documented withdrawal, withholding, or non-escalation of life-sustaining treatment primarily because of adverse neurological prognosis, as recorded in the neonatal unit registry and discharge/death summaries. The registry captured neuro-WWLST only when this process culminated in death; it did not systematically capture neuroprognostic counseling episodes, treatment-limitation discussions, decisions not followed by death, or non-neurologic treatment-limitation pathways.

▪ **Unit context and neuro-WWLST ascertainment.** The study was conducted in a single tertiary neonatal unit with long-standing continuity in neonatal care and routine clinical documentation. Since the 1990s, the unit progressively incorporated family-centered developmental care, parental presence, kangaroo care, breastfeeding support, and comfort-focused care into routine practice. From the 2000s onward, these practices became increasingly formalized through structured developmental-care programs, the human milk bank, and NIDCAP-based training and accreditation. This context is provided to clarify the clinical environment in which major end-of-life decisions were made and documented, not to imply that neuro-WWLST practices, counseling, documentation, or neonatal care remained unchanged across the 30-year study period. In routine care, decisions regarding limitation, withdrawal, or non-escalation of life-sustaining treatment were made through deliberation by the responsible neonatal team, taking into account the infant's overall clinical condition, neurological findings, serial neuroimaging, expected prognosis, comorbidities, clinical evolution, and discussion with the family. The process was not based on a prespecified CUS threshold, and the three-domain imaging framework used in the present study was not used as a decision rule. The registry did not systematically distinguish withdrawal from withholding or non-escalation of life-sustaining treatment because care plans were individualized according to the infant's clinical situation, comfort needs,

treatment tolerance, and family preferences; these situations were therefore analyzed together as neuro-WWLST.

Supplementary Methods S6. Scan-selection rules, robustness and sensitivity analyses, statistical analysis, and missing data.

▪ **Scan-selection definitions: common maximal-burden CUS and last-window CUS.**

▪ **Common maximal-burden CUS.** For cross-outcome comparisons requiring a single per-infant severity summary, maximal-burden CUS was selected using the same prespecified hierarchical rule in all outcome groups: survivors, deaths not classified as neuro-WWLST in the registry, and deaths following documented neuro-WWLST. The hierarchy first maximized the number of severe imaging domains present at the selected scan: parenchymal lesion ≥1 cm, grade 3 IVH, and VM≥2; second, if tied, maximized the joint LP+IVH+VM severity sum; and third, if still tied, selected the earliest CUS window. This common rule was used to improve comparability across outcome groups and was not intended as a clinical decision criterion.

▪ **Last-window CUS.** Among deaths following documented neuro-WWLST, last-window CUS was analyzed separately to describe the outcome-proximal imaging context before death. Last-window CUS was defined as the last recorded CUS within the prespecified surveillance windows before death. It did not need to coincide with the final clinically performed ultrasound before death. For descriptive timing analyses, last-window CUS was assigned to the corresponding analytic window according to acquisition date. Delay of 1 or ≥2 windows therefore reflected progression from the entity-debut window to a later recorded window. Last-window CUS was not used as the primary cross-outcome comparator against survivors or deaths not classified as neuro-WWLST.

▪ **Robustness and sensitivity analyses.** Sensitivity analyses addressed the effect of scan-selection rules and survival-dependent imaging opportunity. First, outcome-group comparisons were performed using the common maximal-burden CUS definition across all outcome groups, with overall and entity-specific panels reported in Supplementary Table S9. Second, among deaths following documented neuro-WWLST, concordance was evaluated between last-window CUS and maximal-burden CUS to

assess whether the outcome-proximal scan captured maximal documented imaging burden (Supplementary Table S11). Third, among survivors and deaths not classified as neuro-WWLST, last available CUS was compared with maximal-burden CUS to assess whether a last-CUS-for-all approach would underestimate maximal documented burden after partial resolution of IVH, parenchymal echogenicity, or ventriculomegaly (Supplementary Table S12). Fourth, fixed early-window CUS1–CUS2 comparisons were performed to describe early documented burden before later survival-dependent imaging opportunities (Supplementary Table S13). Finally, restricted adjusted and sparse-data sensitivity models were fitted where estimable (Supplementary Table S14).

Additional algorithm-resolution profiling and alternative tie-breaking analyses for maximal-burden CUS selection were used as ancillary robustness checks and are reported in Supplementary Tables S25 and S26. These algorithm-specific analyses did not replace the primary cross-outcome comparison, which used the common maximal-burden CUS analysis reported in Supplementary Table S9.

▪ **Statistical analysis.** Data management and cleaning included range checks for GA and BW, verification of permissible LP/IVH/VM grades, and internal chronology checks across CUS windows and dates. Continuous variables are reported as median [IQR] and categorical variables as n/N (%). Analyses were descriptive and exploratory. The primary cross-outcome imaging comparison used the common maximal-burden CUS definition in all in-hospital outcome groups: survivors, deaths not classified as neuro-WWLST in the registry, and deaths following documented neuro-WWLST. Pairwise contrasts focused on deaths following documented neuro-WWLST versus survivors and deaths following documented neuro-WWLST versus deaths not classified as neuro-WWLST. Continuous and ordinal variables were compared using the Mann–Whitney U test. Effect sizes for continuous or ordinal variables are presented as Hodges–Lehmann location shifts, calculated as deaths following documented neuro-WWLST minus the comparator group, with 95% confidence intervals obtained by nonparametric bootstrap using 5,000 resamples. Categorical variables were compared using Fisher's exact test. For binary contrasts, effect sizes are reported as odds ratios with 95% confidence intervals. Haldane–Anscombe correction was applied when any cell count was zero; contrasts with invariant variables or insufficient information for stable estimation were labeled as not estimable. Infinite odds ratios were interpreted as descriptive markers of sparse-data uncertainty. Given the descriptive and exploratory

nature of the analyses, p values were interpreted descriptively and no correction for multiple comparisons was applied.

Entity-specific comparisons were interpreted descriptively because deaths following documented neuro-WWLST were few within individual SBI entities. The forest plot in Figure 4 displays unadjusted descriptive odds ratios derived from the common maximal-burden CUS comparison reported in Supplementary Table S9. These estimates were intended to visualize entity-specific heterogeneity and were not interpreted as predictive, causal, or independently adjusted associations.

Restricted adjusted and sparse-data sensitivity models were fitted only where estimable. For the overall SBI cohort, selected models adjusted for GA, birth epoch, and SBI entity. Entity-specific adjusted models were not emphasized when event counts were sparse, separation occurred, or estimates were unstable. These models were used as sensitivity analyses only and did not replace the primary descriptive comparisons. Birth weight was included in descriptive comparisons but was not included together with GA in restricted adjusted models because of collinearity and sparse event counts. ▪

▪ **Exploratory adjusted survivor outcome models.** Exploratory logistic regression models were fitted to assess whether severe involvement across all three imaging domains was associated with CP and clinically classified school-age cognitive sequelae among survivors. The exposure was severe involvement across all three domains versus fewer than three severe domains, assessed at the maximal-burden CUS. Models were adjusted for gestational age, birth epoch, and SBI entity. Birth weight was not included in the primary adjusted models to avoid collinearity with gestational age. These models were descriptive, interpreted cautiously because of small subgroup sizes, and were not intended for prediction. Cognitive outcome models were additionally interpreted cautiously because cognitive sequelae were clinically classified from heterogeneous longitudinal sources rather than measured as harmonized continuous IQ endpoints.

▪ **Missing data.** The CUS1-imaged analytic denominator and comparison with eligible infants without complete CUS1 are reported in Supplementary Table S1. Scheduled complete CUS-window availability is reported by outcome group and SBI entity in Supplementary Table S10. Additional CUS-window availability and complete three-domain availability across SBI entities are reported in Supplementary Tables S23–S24. Later-window availability was interpreted as outcome- and clinical-course dependent

rather than missing completely at random, particularly among infants who died before the scheduled CUS3 or CUS4 windows. Scan-selection robustness and sensitivity analyses are reported in Supplementary Tables S9 and S11–S14. Topographic descriptors, including laterality and lobar extent, were available only when explicitly reported and are therefore presented with analysis-specific denominators; missingness reflects absent documentation in the clinical report. Neurodevelopmental outcome analyses were restricted to survivors with available follow-up data for each outcome; follow-up availability is summarized in Supplementary Table S18. Analyses were complete-case; no imputation was performed.

Supplementary Methods S7. Neurodevelopmental follow-up and survivor outcome analyses.

▪ **Neurodevelopmental follow-up program.** Neurodevelopmental follow-up was embedded in the longitudinal follow-up program. Children were assessed during infancy, early childhood, and school age according to age and clinical need. The standard program included neurological examination, motor development, developmental assessment, cognitive and educational review, and assessment of functional support needs up to approximately 7 years. Children with CP, clinically classified school-age cognitive sequelae, or other neurological morbidity continued follow-up beyond the standard program when clinically indicated.

▪ **Cerebral palsy.** CP was assessed at approximately 2 years' corrected age and classified by clinical phenotype and functional severity using the Gross Motor Function Classification System (GMFCS). For the present analyses, CP was treated as a binary outcome. Functional severity was summarized as ambulatory CP, defined as GMFCS I–II, and non-ambulatory CP, defined as GMFCS III–V. Denominators excluded survivors with missing CP data. CP motor phenotype was classified from the registry PCI variable among survivors with CP and available phenotype data. Registry categories were grouped descriptively as unilateral/hemiparetic CP for monoplegia or hemiplegia, diplegic/paraparetic CP for paraparesis, triparetic/tetraparetic CP for triparesis or tetraparesis, and other/mixed CP phenotype for spastic-dystonic or ataxic forms. Phenotype-specific analyses were descriptive only because strata were small.

▪ **School-age cognitive outcome.** Clinically classified school-age cognitive sequelae were classified as absent, mild, moderate, or severe from all available longitudinal follow-up information, usually around

the preschool-to-school transition or the 7-year assessment window when children were age-eligible. Available sources included longitudinal developmental and clinical assessments, hospital-based psychometric or neuropsychological testing when available, educational psychology reports, school-based assessments, therapy-related information, school-support documentation, and information on educational functioning. Because the cohort covered three decades, instruments and information sources varied over time according to epoch, age at assessment, clinical indication, language availability, and routine practice. Cognitive outcome was therefore not analyzed as a harmonized continuous IQ score, but as a clinically assigned ordinal severity variable, anchored to approximate developmental/IQ ranges when formal testing was available and supported by educational functioning.

- **No school-age cognitive sequelae** corresponded to cognitive performance within the expected range, broadly equivalent to IQ >84 when formal testing was available.
- **Mild sequelae** corresponded to borderline or low-average cognitive function, broadly equivalent to IQ 71–84, and included mild learning difficulties, school-support needs, or minor curricular adaptation.
- **Moderate sequelae** corresponded to intellectual disability or marked cognitive impairment, broadly equivalent to IQ 55–70, and included substantial educational support, significant curricular adaptation, or special education.
- **Severe sequelae** corresponded to severe cognitive impairment, broadly equivalent to IQ <55, and included severe intellectual disability, very limited functional communication, or severe global developmental disability. These IQ ranges were used as approximate interpretative anchors rather than uniformly measured numerical endpoints.

Source type for school-age cognitive classification was not recorded as a standardized patient-level variable across the full 30-year cohort; therefore, source-stratified analyses comparing formal psychometric testing with broader clinical or educational documentation were not performed. For this reason, analyses involving school-age cognitive sequelae were considered exploratory.

▪ **Exploratory survivor outcome analysis.** Analyses were restricted to survivors within the three SBI entities. Severe imaging domains were parenchymal lesion ≥1 cm, grade 3 IVH, and moderate-to-severe ventriculomegaly, assessed at maximal-burden CUS. The number of severe imaging domains was

summarized as 0, 1, 2, or 3. CP and clinically classified school-age cognitive outcomes were summarized by SBI entity and severe-domain count. Exploratory comparisons across all SBI survivors compared infants with severe involvement across all three imaging domains with those with fewer than three severe domains using Fisher's exact test and adjusted logistic regression models, as detailed in Supplementary Methods S6. Follow-up availability is reported in Supplementary Table S18. Descriptive CP, GMFCS, CP phenotype, and cognitive outcome categories are reported in Supplementary Tables S19–S21, and exploratory adjusted models are reported in Supplementary Table S22.

Supplementary Tables

Index of supplementary tables

1. Supplementary Table S1. Eligible infants with and without complete CUS1
2. Supplementary Table S2. Severe brain injury prevalence by gestational age group in the CUS1-imaged cohort
3. Supplementary Table S3. SBI prevalence in the CUS1-imaged denominator, by decade
4. Supplementary Table S4. Distribution of SBI entities within SBI, by decade
5. Supplementary Table S5. Cohort outcomes by decade in the CUS1-imaged denominator
6. Supplementary Table S6. Cohort outcomes by gestational age group within each decade
7. Supplementary Table S7. Cohort outcomes by gestational age group in the CUS1-imaged denominator
8. Supplementary Table S8. SBI entities, outcome distribution, and within-entity subgroups defined by coexisting grade 3 IVH or additional parenchymal injury
9. Supplementary Table S9. Outcome-group comparisons using common maximal-burden CUS
10. Supplementary Table S10. Scheduled complete CUS-window availability by outcome group and SBI entity
11. Supplementary Table S11. Concordance between last-window CUS and maximal-burden CUS among neuro-WWLST deaths
12. Supplementary Table S12. Last available CUS versus maximal-burden CUS among survivors and deaths not classified as neuro-WWLST
13. Supplementary Table S13. Fixed early-window CUS1–CUS2 imaging burden
14. Supplementary Table S14. Restricted adjusted and sparse-data sensitivity models
15. Supplementary Table S15. PVHI: gestational age and neuro-WWLST by coexisting grade 3 IVH, overall and by debut window
16. Supplementary Table S16. IVH3 entity: gestational age and neuro-WWLST by additional parenchymal injury versus isolated IVH3
17. Supplementary Table S17. cPVL: gestational age and neuro-WWLST by coexisting grade 3 IVH, overall and by debut window
18. Supplementary Table S18. Neurodevelopmental follow-up availability among SBI survivors
19. Supplementary Table S19. Cerebral palsy and GMFCS among survivors by SBI entity and severe-domain count
20. Supplementary Table S20. Cerebral palsy motor phenotype among survivors with CP by SBI entity and severe-domain count
21. Supplementary Table S21. Clinically classified school-age cognitive sequelae among survivors by SBI entity and severe-domain count
22. Supplementary Table S22. Exploratory adjusted associations between severe three-domain involvement and survivor outcomes
23. Supplementary Table S23. Scheduled CUS window availability across SBI entities
24. Supplementary Table S24. Complete three-domain availability across SBI entities
25. Supplementary Table S25. Algorithm-resolution profile for maximal-burden CUS selection in PVHI without neuro-WWLST
26. Supplementary Table S26. Sensitivity to alternative tie-breaking rules for maximal-burden CUS selection in PVHI without neuro-WWLST

Supplementary Table S1. Eligible infants with and without complete CUS1

Variable	All eligible infants	CUS1-imaged cohort	No recorded complete CUS1
n	3081	2841	240
GA, weeks, median [IQR]	29 [27–31]	29 [27–31]	25 [23–29]
Birth weight, g, median [IQR]	1100 [830–1350]	1130 [865–1351]	667 [557.5–1101.5]
Male sex, n/N (%)	1627/3081 (52.8)	1481/2841 (52.1)	146/240 (60.8)
Born in study center, n/N (%)	2828/3081 (91.8)	2616/2841 (92.1)	212/240 (88.3)
Multiple gestation, n/N (%)	933/3081 (30.3)	874/2841 (30.8)	59/240 (24.6)
1991–2000, n/N (%)	856/3081 (27.8)	752/2841 (26.5)	104/240 (43.3)
2001–2010, n/N (%)	933/3081 (30.3)	867/2841 (30.5)	66/240 (27.5)
2011–2020, n/N (%)	1292/3081 (41.9)	1222/2841 (43.0)	70/240 (29.2)
Death before 2 years', n/N (%)	512/3081 (16.6)	309/2841 (10.9)	203/240 (84.6)
Death not classified as neuro-WWLST, n/N (%)	467/3081 (15.2)	264/2841 (9.3)	203/240 (84.6)
Neuro-WWLST death, n/N (%)	45/3081 (1.5)	45/2841 (1.6)	0/240 (0.0)
Death in delivery room/paritorio, n/N (%)	113/3081 (3.7)	0/2841 (0.0)	113/240 (47.1)
At least one complete later CUS (if no CUS1), n/N (%)	32/3081 (1.0)	0/2841 (0.0)	32/240 (13.3)
No complete CUS window, n/N (%)	208/3081 (6.8)	0/2841 (0.0)	208/240 (86.7)

Note. Complete CUS1 was defined as availability of all three prespecified domains—IVH grade, parenchymal lesion grade and VM grade—within the first 48-hour/day-2 window. Infants without complete CUS1 were not included in the CUS1-imaged analytic cohort.

Supplementary Table S2. Severe brain injury prevalence by gestational age group in the CUS1-imaged cohort

GA group (weeks)	N	PVHI, n (%)	IVH3 entity, n (%)	cPVL, n (%)	Total SBI, n (%)
22–24	180	19 (10.6%)	16 (8.9%)	5 (2.8%)	40 (22.2%)
25–26	435	42 (9.7%)	27 (6.2%)	18 (4.1%)	87 (20.0%)
27–28	610	23 (3.8%)	29 (4.8%)	21 (3.4%)	73 (12.0%)
≥29	1,616	33 (2.0%)	22 (1.4%)	30 (1.9%)	85 (5.3%)
Overall	2,841	117 (4.1%)	94 (3.3%)	75 (2.6%)	286 (10.1%)

Note. Denominator is infants with complete CUS1. Percentages are calculated within gestational age stratum. SBI comprised the mutually exclusive entities PVHI, IVH3 entity and cPVL.

Supplementary Table S3. SBI prevalence in the CUS1-imaged denominator, by decade

Decade	N with complete CUS1	PVHI	IVH3 entity	cPVL	Total SBI
1991–2000	752	32 (4.3%)	30 (4.0%)	32 (4.3%)	94 (12.5%)
2001–2010	867	23 (2.7%)	42 (4.8%)	21 (2.4%)	86 (9.9%)
2011–2020	1,222	62 (5.1%)	22 (1.8%)	22 (1.8%)	106 (8.7%)
Overall	2,841	117 (4.1%)	94 (3.3%)	75 (2.6%)	286 (10.1%)

Note. Denominator is infants with complete CUS1 within each decade; cells are n (%) within decade. SBI entities were mutually exclusive.

Supplementary Table S4. Distribution of SBI entities within SBI, by decade

Decade	Total SBI (n)	PVHI	IVH3 entity	cPVL
1991–2000	94	32 (34.0%)	30 (31.9%)	32 (34.0%)
2001–2010	86	23 (26.7%)	42 (48.8%)	21 (24.4%)
2011–2020	106	62 (58.5%)	22 (20.8%)	22 (20.8%)
Overall	286	117 (40.9%)	94 (32.9%)	75 (26.2%)

Note. Rows are restricted to infants with SBI within each decade; cells are n (%) within decade. SBI entities were mutually exclusive.

Supplementary Table S5. Cohort outcomes by decade in the CUS1-imaged denominator

Decade	N	GA, weeks, median [IQR]	Survived, n (%)	Death not classified as neuro-WWLST, n (%)	Neuro-WWLST death, n (%)
1991–2000	752	29 [27–31]	658 (87.5%)	83 (11.0%)	11 (1.5%)
2001–2010	867	29 [27–31]	744 (85.8%)	111 (12.8%)	12 (1.4%)
2011–2020	1,222	29 [27–31]	1,130 (92.5%)	70 (5.7%)	22 (1.8%)
Overall	2,841	29 [27–31]	2,532 (89.1%)	264 (9.3%)	45 (1.6%)

Note. Denominator is the CUS1-imaged cohort. Percentages are within decade. Outcomes were survival to discharge, death not classified as neuro-WWLST, and neuro-WWLST death.

Supplementary Table S6. Cohort outcomes by gestational age group within each decade

Panel A. 1991–2000

GA group (weeks)	N	Survived, n (%)	Death not classified as neuro-WWLST, n (%)	Neuro-WWLST death, n (%)
22–24	38	18 (47.4%)	19 (50.0%)	1 (2.6%)
25–26	105	70 (66.7%)	26 (24.8%)	9 (8.6%)
27–28	163	145 (89.0%)	17 (10.4%)	1 (0.6%)
29–30	199	190 (95.5%)	9 (4.5%)	0 (0.0%)
≥31	247	235 (95.1%)	12 (4.9%)	0 (0.0%)

Panel B. 2001–2010

GA group (weeks)	N	Survived, n (%)	Death not classified as neuro-WWLST, n (%)	Neuro-WWLST death, n (%)
22–24	56	29 (51.8%)	24 (42.9%)	3 (5.4%)
25–26	149	114 (76.5%)	31 (20.8%)	4 (2.7%)
27–28	196	170 (86.7%)	23 (11.7%)	3 (1.5%)
29–30	210	192 (91.4%)	17 (8.1%)	1 (0.5%)
≥31	256	239 (93.4%)	16 (6.2%)	1 (0.4%)

Panel C. 2011–2020

GA group (weeks)	N	Survived, n (%)	Death not classified as neuro-WWLST, n (%)	Neuro-WWLST death, n (%)
22–24	86	60 (69.8%)	19 (22.1%)	7 (8.1%)
25–26	181	158 (87.3%)	14 (7.7%)	9 (5.0%)
27–28	251	229 (91.2%)	20 (8.0%)	2 (0.8%)
29–30	351	339 (96.6%)	10 (2.8%)	2 (0.6%)
≥31	353	344 (97.5%)	7 (2.0%)	2 (0.6%)

Note. Denominator is infants with complete CUS1 within each decade. Percentages are within gestational age stratum.

Supplementary Table S7. Cohort outcomes by gestational age group in the CUS1-imaged denominator

GA group (weeks)	N	Survived, n (%)	Death not classified as neuro-WWLST, n (%)	Neuro-WWLST death, n (%)
22–24	180	107 (59.4%)	62 (34.4%)	11 (6.1%)
25–26	435	342 (78.6%)	71 (16.3%)	22 (5.1%)
27–28	610	544 (89.2%)	60 (9.8%)	6 (1.0%)
≥29	1,616	1,539 (95.2%)	71 (4.4%)	6 (0.4%)

Note. Denominator is the CUS1-imaged cohort. Percentages are within gestational age group.

Supplementary Table S8. SBI entities, outcome distribution, and within-entity subgroups defined by coexisting grade 3 IVH or additional parenchymal injury

SBI entity / subgroup	n	n (% CUS1-imaged cohort) (n=2,841)	n (% of SBI) (n=286)	Survived, n/N (%)	Death not classified as neuro-WWLST, n/N (%)	Neuro-WWLST deaths, n/N (%)	Share of SBI neuro-WWLST deaths, n/N (%) (n=43)
SBI (total)	286	10.1%	100%	197/286 (68.9%)	46/286 (16.1%)	43/286 (15.0%)	43/43 (100%)
PVHI (total)	117	117/2,841 (4.1%)	117/286 (40.9%)	75/117 (64.1%)	18/117 (15.4%)	24/117 (20.5%)	24/43 (55.8%)
PVHI (with maximal IVH <3)	61	61/2,841 (2.1%)	61/286 (21.3%)	48/61 (78.7%)	9/61 (14.8%)	4/61 (6.6%)	4/43 (9.3%)
PVHI (with coexisting grade 3 IVH)	56	56/2,841 (2.0%)	56/286 (19.6%)	27/56 (48.2%)	9/56 (16.1%)	20/56 (35.7%)	20/43 (46.5%)
IVH3 entity (total)	94	94/2,841 (3.3%)	94/286 (32.9%)	66/94 (70.2%)	19/94 (20.2%)	9/94 (9.6%)	9/43 (20.9%)
Isolated IVH3	86	86/2,841 (3.0%)	86/286 (30.1%)	64/86 (74.4%)	17/86 (19.8%)	5/86 (5.8%)	5/43 (11.6%)
IVH3 entity with additional parenchymal injury*	8	8/2,841 (0.3%)	8/286 (2.8%)	2/8 (25.0%)	2/8 (25.0%)	4/8 (50.0%)	4/43 (9.3%)
cPVL (total)	75	75/2,841 (2.6%)	75/286 (26.2%)	56/75 (74.7%)	9/75 (12.0%)	10/75 (13.3%)	10/43 (23.3%)
cPVL (with maximal IVH <3)	59	59/2,841 (2.1%)	59/286 (20.6%)	47/59 (79.7%)	4/59 (6.8%)	8/59 (13.6%)	8/43 (18.6%)
cPVL (with coexisting grade 3 IVH)	16	16/2,841 (0.6%)	16/286 (5.6%)	9/16 (56.3%)	5/16 (31.3%)	2/16 (12.5%)	2/43 (4.7%)

Note. Values are n/N (%) unless otherwise stated. Cohort percentages use the CUS1-imaged denominator (n=2,841), and SBI percentages use all SBI cases (n=286). SBI entities were mutually exclusive by design. Within PVHI and cPVL, subgroups were defined by maximal coexisting IVH severity (<3 versus grade 3). Within the IVH3 entity, subgroups were defined as isolated IVH3 or IVH3 with additional parenchymal injury. *Additional parenchymal injury included arterial infarction/hemorrhage, either lobar or cerebellar, and mixed focal parenchymal lesions with cystic and hemorrhagic features that did not fulfill PVHI or cPVL criteria.

Supplementary Table S9. Outcome-group comparisons using common maximal-burden CUS

Panel A. All SBI

Variable	Survivors	Death not classified as neuro-WWLST	Neuro-WWLST death	Neuro-WWLST vs survivors	Neuro-WWLST vs Death not classified as neuro-WWLST
All SBI					
n	197	46	43		
Gestational age, weeks, median [IQR]	28 [26–29]	26 [25–29]	26 [24.5–26.5]	p<0.001	p=0.094
Birth weight, g, median [IQR]	1010 [830–1230]	855 [675–1084.8]	775 [663–985]	p<0.001	p=0.435
Birth epoch 1991–2000	61/197 (31.0)	22/46 (47.8)	11/43 (25.6)	0.77 (0.36–1.62); p=0.583	0.38 (0.15–0.92); p=0.047
Birth epoch 2001–2010	58/197 (29.4)	17/46 (37.0)	11/43 (25.6)	0.82 (0.39–1.74); p=0.712	0.59 (0.24–1.46); p=0.264
Birth epoch 2011–2020	78/197 (39.6)	7/46 (15.2)	21/43 (48.8)	1.46 (0.75–2.83); p=0.306	5.32 (1.95–14.49); p=0.001
SBI entity: PVHI	75/197 (38.1)	18/46 (39.1)	24/43 (55.8)	2.05 (1.05–4.00); p=0.040	1.96 (0.84–4.57); p=0.140
SBI entity: IVH3 entity	66/197 (33.5)	19/46 (41.3)	9/43 (20.9)	0.53 (0.24–1.16); p=0.146	0.38 (0.15–0.96); p=0.043
SBI entity: cPVL	56/197 (28.4)	9/46 (19.6)	10/43 (23.3)	0.76 (0.35–1.65); p=0.574	1.25 (0.45–3.44); p=0.797
Maximal-burden CUS selected at CUS1	30/197 (15.2)	17/46 (37.0)	14/43 (32.6)	2.69 (1.27–5.67); p=0.015	0.82 (0.34–1.98); p=0.824
Maximal-burden CUS selected at CUS2	39/197 (19.8)	18/46 (39.1)	19/43 (44.2)	3.21 (1.60–6.44); p=0.001	1.23 (0.53–2.86); p=0.671
Maximal-burden CUS selected at CUS3	88/197 (44.7)	9/46 (19.6)	7/43 (16.3)	0.24 (0.10–0.57); p<0.001	0.80 (0.27–2.38); p=0.786
Maximal-burden CUS selected at CUS4	40/197 (20.3)	2/46 (4.3)	3/43 (7.0)	0.29 (0.09–1.00); p=0.047	1.65 (0.26–10.39); p=0.670
Maximal-burden CUS in CUS1–CUS2	69/197 (35.0)	35/46 (76.1)	33/43 (76.7)	6.12 (2.85–13.16); p<0.001	1.04 (0.39–2.76); p=1.000
Parenchymal lesion ≥1 cm (LP4)	90/197 (45.7)	18/46 (39.1)	35/43 (81.4)	5.20 (2.30–11.78); p<0.001	6.81 (2.58–17.95); p<0.001
Grade 3 IVH	97/197 (49.2)	33/46 (71.7)	31/43 (72.1)	2.66 (1.29–5.49); p=0.007	1.02 (0.40–2.57); p=1.000
VM≥2	79/197 (40.1)	10/46 (21.7)	22/43 (51.2)	1.56 (0.81–3.03); p=0.233	3.77 (1.50–9.47); p=0.005
≥2 severe domains	84/197 (42.6)	16/46 (34.8)	30/43 (69.8)	3.10 (1.53–6.31); p=0.001	4.33 (1.78–10.53); p=0.001
Severe three-domain involvement	17/197 (8.6)	3/46 (6.5)	16/43 (37.2)	6.27 (2.84–13.87); p<0.001	8.49 (2.26–31.91); p<0.001
Posthemorrhagic hydrocephalus	30/197 (15.2)	0/46 (0.0)	7/43 (16.3)	1.08 (0.44–2.66); p=0.819	∞ (1.06–345.70); p=0.005
VP shunt placement	17/197 (8.6)	0/46 (0.0)	0/43 (0.0)	0.00 (0.01–2.01); p=0.048	Not estimable; p=1.000

Panel B. PVHI

Variable	Survivors	Death not classified as neuro-WWLST	Neuro-WWLST death	Neuro-WWLST vs survivors	Neuro-WWLST vs Death not classified as neuro-WWLST
PVHI					
n	75	18	24		

Variable	Survivors	Death not classified as neuro-WWLST	Neuro-WWLST death	Neuro-WWLST vs survivors	Neuro-WWLST vs Death not classified as neuro-WWLST
Gestational age, weeks, median [IQR]	27 [25.5–29]	26 [24.2–27]	25.5 [25–26]	p=0.006	p=0.438
Birth weight, g, median [IQR]	950 [800–1170.5]	800 [675–907.5]	812.5 [669.2–965]	p=0.011	p=1.000
Birth epoch 1991–2000	18/75 (24.0)	9/18 (50.0)	5/24 (20.8)	0.83 (0.27–2.55); p=1.000	0.26 (0.07–1.02); p=0.096
Birth epoch 2001–2010	12/75 (16.0)	5/18 (27.8)	6/24 (25.0)	1.75 (0.58–5.32); p=0.366	0.87 (0.22–3.46); p=1.000
Birth epoch 2011–2020	45/75 (60.0)	4/18 (22.2)	13/24 (54.2)	0.79 (0.31–1.99); p=0.641	4.14 (1.05–16.29); p=0.057
Maximal-burden CUS selected at CUS1	18/75 (24.0)	9/18 (50.0)	9/24 (37.5)	1.90 (0.71–5.07); p=0.201	0.60 (0.17–2.07); p=0.533
Maximal-burden CUS selected at CUS2	17/75 (22.7)	5/18 (27.8)	13/24 (54.2)	4.03 (1.53–10.61); p=0.005	3.07 (0.83–11.36); p=0.120
Maximal-burden CUS selected at CUS3	33/75 (44.0)	3/18 (16.7)	2/24 (8.3)	0.12 (0.03–0.53); p=0.001	0.45 (0.07–3.06); p=0.636
Maximal-burden CUS selected at CUS4	7/75 (9.3)	1/18 (5.6)	0/24 (0.0)	0.00 (0.01–3.39); p=0.190	0.00 (0.01–6.20); p=0.429
Maximal-burden CUS in CUS1–CUS2	35/75 (46.7)	14/18 (77.8)	22/24 (91.7)	12.57 (2.76–57.31); p<0.001	3.14 (0.51–19.49); p=0.375
Parenchymal lesion ≥1 cm (LP4)	40/75 (53.3)	10/18 (55.6)	22/24 (91.7)	9.62 (2.11–43.87); p<0.001	8.80 (1.58–49.16); p=0.010
Grade 3 IVH	26/75 (34.7)	9/18 (50.0)	20/24 (83.3)	9.42 (2.91–30.49); p<0.001	5.00 (1.21–20.61); p=0.041
VM≥2	24/75 (32.0)	2/18 (11.1)	14/24 (58.3)	2.98 (1.16–7.66); p=0.030	11.20 (2.09–60.04); p=0.003
≥2 severe domains	27/75 (36.0)	5/18 (27.8)	21/24 (87.5)	12.44 (3.40–45.59); p<0.001	18.20 (3.71–89.23); p<0.001
Severe three-domain involvement	11/75 (14.7)	1/18 (5.6)	12/24 (50.0)	5.82 (2.09–16.21); p=0.001	17.00 (1.94–148.85); p=0.002
Posthemorrhagic hydrocephalus	9/75 (12.0)	0/18 (0.0)	1/24 (4.2)	0.32 (0.04–2.66); p=0.443	∞ (0.09–61.41); p=1.000
VP shunt placement	6/75 (8.0)	0/18 (0.0)	0/24 (0.0)	0.00 (0.01–4.02); p=0.331	Not estimable; p=1.000

Panel C. IVH3 entity

Variable	Survivors	Death not classified as neuro-WWLST	Neuro-WWLST death	Neuro-WWLST vs survivors	Neuro-WWLST vs Death not classified as neuro-WWLST
IVH3 entity					
n	66	19	9		
Gestational age, weeks, median [IQR]	27 [26–28]	26 [24.5–29]	25 [25–26]	p=0.026	p=0.370
Birth weight, g, median [IQR]	1085 [851.2–1237.5]	860 [738.5–1177.5]	750 [650–850]	p=0.002	p=0.192
Birth epoch 1991–2000	19/66 (28.8)	8/19 (42.1)	3/9 (33.3)	1.24 (0.28–5.46); p=0.716	0.69 (0.13–3.61); p=1.000
Birth epoch 2001–2010	29/66 (43.9)	9/19 (47.4)	4/9 (44.4)	1.02 (0.25–4.15); p=1.000	0.89 (0.18–4.37); p=1.000
Birth epoch 2011–2020	18/66 (27.3)	2/19 (10.5)	2/9 (22.2)	0.76 (0.14–4.02); p=1.000	2.43 (0.28–20.82); p=0.574
Maximal-burden CUS selected at CUS1	9/66 (13.6)	6/19 (31.6)	3/9 (33.3)	3.17 (0.67–14.98); p=0.151	1.08 (0.20–5.87); p=1.000
Maximal-burden CUS selected at CUS2	20/66 (30.3)	12/19 (63.2)	4/9 (44.4)	1.84 (0.45–7.58); p=0.455	0.47 (0.09–2.34); p=0.432

Variable	Survivors	Death not classified as neuro-WWLST	Neuro-WWLST death	Neuro-WWLST vs survivors	Neuro-WWLST vs Death not classified as neuro-WWLST
Maximal-burden CUS selected at CUS3	30/66 (45.5)	1/19 (5.3)	2/9 (22.2)	0.34 (0.07–1.78); p=0.286	5.14 (0.40–66.15); p=0.234
Maximal-burden CUS selected at CUS4	7/66 (10.6)	0/19 (0.0)	0/9 (0.0)	0.00 (0.02–7.93); p=0.588	Not estimable; p=1.000
Maximal-burden CUS in CUS1–CUS2	29/66 (43.9)	18/19 (94.7)	7/9 (77.8)	4.47 (0.86–23.13); p=0.079	0.19 (0.02–2.50); p=0.234
Parenchymal lesion ≥1 cm (LP4)	3/66 (4.5)	2/19 (10.5)	3/9 (33.3)	10.50 (1.72–63.92); p=0.020	4.25 (0.57–31.94); p=0.290
Grade 3 IVH	65/66 (98.5)	19/19 (100.0)	9/9 (100.0)	Not estimated; p=1.000	Not estimable; p=1.000
VM≥2	42/66 (63.6)	6/19 (31.6)	5/9 (55.6)	0.71 (0.17–2.92); p=0.720	2.71 (0.53–13.86); p=0.409
≥2 severe domains	42/66 (63.6)	7/19 (36.8)	6/9 (66.7)	1.14 (0.26–4.99); p=1.000	3.43 (0.65–18.22); p=0.228
Severe three-domain involvement	2/66 (3.0)	1/19 (5.3)	2/9 (22.2)	9.14 (1.11–75.38); p=0.068	5.14 (0.40–66.15); p=0.234
Posthemorrhagic hydrocephalus	14/66 (21.2)	0/19 (0.0)	4/9 (44.4)	2.97 (0.70–12.56); p=0.205	∞ (1.48–688.22); p=0.006
VP shunt placement	7/66 (10.6)	0/19 (0.0)	0/9 (0.0)	0.00 (0.02–7.93); p=0.588	Not estimable; p=1.000

Panel D. cPVL

Variable	Survivors	Death not classified as neuro-WWLST	Neuro-WWLST death	Neuro-WWLST vs survivors	Neuro-WWLST vs Death not classified as neuro-WWLST
cPVL					
n	56	9	10		
Gestational age, weeks, median [IQR]	28 [27–30]	28 [26–30]	26 [24–29.5]	p=0.074	p=0.184
Birth weight, g, median [IQR]	1052 [850–1260]	998 [620–1100]	870 [662.5–1197.5]	p=0.186	p=0.713
Birth epoch 1991–2000	24/56 (42.9)	5/9 (55.6)	3/10 (30.0)	0.57 (0.13–2.44); p=0.508	0.34 (0.05–2.26); p=0.370
Birth epoch 2001–2010	17/56 (30.4)	3/9 (33.3)	1/10 (10.0)	0.25 (0.03–2.17); p=0.264	0.22 (0.02–2.67); p=0.303
Birth epoch 2011–2020	15/56 (26.8)	1/9 (11.1)	6/10 (60.0)	4.10 (1.01–16.57); p=0.062	12.00 (1.05–136.80); p=0.057
Maximal-burden CUS selected at CUS1	3/56 (5.4)	2/9 (22.2)	2/10 (20.0)	4.42 (0.64–30.66); p=0.162	0.88 (0.10–7.95); p=1.000
Maximal-burden CUS selected at CUS2	2/56 (3.6)	1/9 (11.1)	2/10 (20.0)	6.75 (0.83–54.90); p=0.106	2.00 (0.15–26.74); p=1.000
Maximal-burden CUS selected at CUS3	25/56 (44.6)	5/9 (55.6)	3/10 (30.0)	0.53 (0.12–2.27); p=0.498	0.34 (0.05–2.26); p=0.370
Maximal-burden CUS selected at CUS4	26/56 (46.4)	1/9 (11.1)	3/10 (30.0)	0.49 (0.12–2.11); p=0.493	3.43 (0.29–40.95); p=0.582
Maximal-burden CUS in CUS1–CUS2	5/56 (8.9)	3/9 (33.3)	4/10 (40.0)	6.80 (1.42–32.47); p=0.024	1.33 (0.20–8.71); p=1.000
Parenchymal lesion ≥1 cm (LP4)	47/56 (83.9)	6/9 (66.7)	10/10 (100.0)	4.20 (0.23–77.98); p=0.334	11.31 (0.50–256.21); p=0.087
Grade 3 IVH	6/56 (10.7)	5/9 (55.6)	2/10 (20.0)	2.08 (0.36–12.18); p=0.596	0.20 (0.03–1.53); p=0.170
VM≥2	13/56 (23.2)	2/9 (22.2)	3/10 (30.0)	1.42 (0.32–6.28); p=0.695	1.50 (0.19–11.93); p=1.000
≥2 severe domains	15/56 (26.8)	4/9 (44.4)	3/10 (30.0)	1.17 (0.27–5.13); p=1.000	0.54 (0.08–3.53); p=0.650

Variable	Survivors	Death not classified as neuro-WWLST	Neuro-WWLST death	Neuro-WWLST vs survivors	Neuro-WWLST vs Death not classified as neuro-WWLST
Severe three-domain involvement	4/56 (7.1)	1/9 (11.1)	2/10 (20.0)	3.25 (0.51–20.74); p=0.222	2.00 (0.15–26.74); p=1.000
Posthemorrhagic hydrocephalus	7/56 (12.5)	0/9 (0.0)	2/10 (20.0)	1.75 (0.31–9.97); p=0.616	∞ (0.23–133.61); p=0.474
VP shunt placement	4/56 (7.1)	0/9 (0.0)	0/10 (0.0)	0.00 (0.03–11.11); p=1.000	Not estimable; p=1.000

Note. Values are n/N (%) unless otherwise indicated. Maximal-burden CUS was selected using the same prespecified hierarchical rule in all outcome groups. Odds ratios are unadjusted descriptive estimates. Infinite ORs reflect zero cells and should be interpreted as sparse-data uncertainty.

Supplementary Table S10. Scheduled complete CUS-window availability by outcome group and SBI entity

Panel A. All SBI

CUS availability	All SBI - Survivors	All SBI - Death not classified as neuro-WWLST	All SBI - Neuro-WWLST death
n	197	46	43
Complete CUS1 (≤48h/day 2)	197/197 (100.0)	46/46 (100.0)	43/43 (100.0)
Complete CUS2 (~day 7)	194/197 (98.5)	33/46 (71.7)	30/43 (69.8)
Complete CUS3 (~day 28)	191/197 (97.0)	17/46 (37.0)	11/43 (25.6)
Complete CUS4 (TEA/discharge)	193/197 (98.0)	8/46 (17.4)	6/43 (14.0)
At least two complete CUS windows	197/197 (100.0)	33/46 (71.7)	30/43 (69.8)
All four complete CUS windows	186/197 (94.4)	7/46 (15.2)	6/43 (14.0)
Only one complete CUS window	0/197 (0.0)	13/46 (28.3)	13/43 (30.2)
Death before CUS3 window	0/197 (0.0)	29/46 (63.0)	32/43 (74.4)
Death before CUS4 window	0/197 (0.0)	38/46 (82.6)	37/43 (86.0)

Panel B. PVHI

CUS availability	PVHI - Survivors	PVHI - Death not classified as neuro-WWLST	PVHI - Neuro-WWLST death
n	75	18	24
Complete CUS1 (≤48h/day 2)	75/75 (100.0)	18/18 (100.0)	24/24 (100.0)
Complete CUS2 (~day 7)	75/75 (100.0)	10/18 (55.6)	15/24 (62.5)
Complete CUS3 (~day 28)	73/75 (97.3)	4/18 (22.2)	3/24 (12.5)
Complete CUS4 (TEA/discharge)	73/75 (97.3)	2/18 (11.1)	1/24 (4.2)
At least two complete CUS windows	75/75 (100.0)	10/18 (55.6)	15/24 (62.5)
All four complete CUS windows	71/75 (94.7)	1/18 (5.6)	1/24 (4.2)
Only one complete CUS window	0/75 (0.0)	8/18 (44.4)	9/24 (37.5)
Death before CUS3 window	0/75 (0.0)	14/18 (77.8)	21/24 (87.5)
Death before CUS4 window	0/75 (0.0)	16/18 (88.9)	23/24 (95.8)

Panel C. IVH3 entity

CUS availability	IVH3 entity - Survivors	IVH3 entity - Death not classified as neuro-WWLST	IVH3 entity - Neuro-WWLST death
n	66	19	9
Complete CUS1 (≤48h/day 2)	66/66 (100.0)	19/19 (100.0)	9/9 (100.0)
Complete CUS2 (~day 7)	65/66 (98.5)	15/19 (78.9)	6/9 (66.7)
Complete CUS3 (~day 28)	63/66 (95.5)	7/19 (36.8)	2/9 (22.2)

CUS availability	IVH3 entity - Survivors	IVH3 entity - Death not classified as neuro-WWLST	IVH3 entity - Neuro-WWLST death
Complete CUS4 (TEA/discharge)	64/66 (97.0)	4/19 (21.1)	2/9 (22.2)
At least two complete CUS windows	66/66 (100.0)	15/19 (78.9)	6/9 (66.7)
All four complete CUS windows	62/66 (93.9)	4/19 (21.1)	2/9 (22.2)
Only one complete CUS window	0/66 (0.0)	4/19 (21.1)	3/9 (33.3)
Death before CUS3 window	0/66 (0.0)	12/19 (63.2)	7/9 (77.8)
Death before CUS4 window	0/66 (0.0)	15/19 (78.9)	7/9 (77.8)

Panel D. cPVL

CUS availability	cPVL - Survivors	cPVL - Death not classified as neuro-WWLST	cPVL - Neuro-WWLST death
n	56	9	10
Complete CUS1 (≤48h/day 2)	56/56 (100.0)	9/9 (100.0)	10/10 (100.0)
Complete CUS2 (~day 7)	54/56 (96.4)	8/9 (88.9)	9/10 (90.0)
Complete CUS3 (~day 28)	55/56 (98.2)	6/9 (66.7)	6/10 (60.0)
Complete CUS4 (TEA/discharge)	56/56 (100.0)	2/9 (22.2)	3/10 (30.0)
At least two complete CUS windows	56/56 (100.0)	8/9 (88.9)	9/10 (90.0)
All four complete CUS windows	53/56 (94.6)	2/9 (22.2)	3/10 (30.0)
Only one complete CUS window	0/56 (0.0)	1/9 (11.1)	1/10 (10.0)
Death before CUS3 window	0/56 (0.0)	3/9 (33.3)	4/10 (40.0)
Death before CUS4 window	0/56 (0.0)	7/9 (77.8)	7/10 (70.0)

Note. All infants in the SBI analytic cohort had complete CUS1. Later-window availability reflects subsequent survival, transfer/discharge and clinical course.

Supplementary Table S11. Concordance between last-window CUS and maximal-burden CUS among neuro-WWLST deaths

Group	n	Last-window CUS contained maximal burden	Same selected maximal-burden window	Same severe-domain count	Same severe three-domain status	Last-window lower burden	Maximal-burden selected earlier due to tie-breaker
All SBI neuro-WWLST deaths	43	43/43 (100.0)	38/43 (88.4)	43/43 (100.0)	43/43 (100.0)	0/43 (0.0)	5/43 (11.6)
PVHI	24	24/24 (100.0)	22/24 (91.7)	24/24 (100.0)	24/24 (100.0)	0/24 (0.0)	2/24 (8.3)
IVH3 entity	9	9/9 (100.0)	7/9 (77.8)	9/9 (100.0)	9/9 (100.0)	0/9 (0.0)	2/9 (22.2)
cPVL	10	10/10 (100.0)	9/10 (90.0)	10/10 (100.0)	10/10 (100.0)	0/10 (0.0)	1/10 (10.0)

Note. Last-window CUS was the last recorded CUS within the prespecified windows before neuro-WWLST death. Maximal-burden CUS was selected using the common hierarchical rule.

Supplementary Table S12. Last available CUS versus maximal-burden CUS among survivors and deaths not classified as neuro-WWLST

Group	n	Variable	Maximal-burden CUS	Last available CUS	Difference (last – maximal, percentage points)
All SBI non-neuro-WWLST	243	Last available lower burden than maximal CUS	0/243 (0.0)	97/243 (39.9)	
All SBI non-neuro-WWLST	243	Severe-domain count, median [IQR]	1 [1–2]	1 [0–2]	
All SBI non-neuro-WWLST	243	Parenchymal lesion ≥1 cm (LP4)	108/243 (44.4)	101/243 (41.6)	-2.9
All SBI non-neuro-WWLST	243	Grade 3 IVH	130/243 (53.5)	59/243 (24.3)	-29.2
All SBI non-neuro-WWLST	243	VM≥2	89/243 (36.6)	76/243 (31.3)	-5.3
All SBI non-neuro-WWLST	243	≥2 severe domains	100/243 (41.2)	62/243 (25.5)	-15.6
All SBI non-neuro-WWLST	243	Severe three-domain involvement	20/243 (8.2)	9/243 (3.7)	-4.5
All SBI non-neuro-WWLST	243	Selected CUS in CUS1–CUS2	104/243 (42.8)	30/243 (12.3)	-30.5
All SBI non-neuro-WWLST	243	Selected CUS in CUS3–CUS4	139/243 (57.2)	213/243 (87.7)	+30.5
PVHI non-neuro-WWLST	93	Last available lower burden than maximal CUS	0/93 (0.0)	36/93 (38.7)	
PVHI non-neuro-WWLST	93	Severe-domain count, median [IQR]	1 [0–2]	1 [0–1]	
PVHI non-neuro-WWLST	93	Parenchymal lesion ≥1 cm (LP4)	50/93 (53.8)	45/93 (48.4)	-5.4
PVHI non-neuro-WWLST	93	Grade 3 IVH	35/93 (37.6)	17/93 (18.3)	-19.4
PVHI non-neuro-WWLST	93	VM≥2	26/93 (28.0)	23/93 (24.7)	-3.2
PVHI non-neuro-WWLST	93	≥2 severe domains	32/93 (34.4)	22/93 (23.7)	-10.8
PVHI non-neuro-WWLST	93	Severe three-domain involvement	12/93 (12.9)	5/93 (5.4)	-7.5
PVHI non-neuro-WWLST	93	Selected CUS in CUS1–CUS2	49/93 (52.7)	13/93 (14.0)	-38.7
PVHI non-neuro-WWLST	93	Selected CUS in CUS3–CUS4	44/93 (47.3)	80/93 (86.0)	+38.7
IVH3 entity non-neuro-WWLST	85	Last available lower burden than maximal CUS	0/85 (0.0)	49/85 (57.6)	
IVH3 entity non-neuro-WWLST	85	Severe-domain count, median [IQR]	2 [1–2]	1 [0–2]	
IVH3 entity non-neuro-WWLST	85	Parenchymal lesion ≥1 cm (LP4)	5/85 (5.9)	5/85 (5.9)	+0.0
IVH3 entity non-neuro-WWLST	85	Grade 3 IVH	84/85 (98.8)	36/85 (42.4)	-56.5
IVH3 entity non-neuro-WWLST	85	VM≥2	48/85 (56.5)	39/85 (45.9)	-10.6
IVH3 entity non-neuro-WWLST	85	≥2 severe domains	49/85 (57.6)	24/85 (28.2)	-29.4
IVH3 entity non-neuro-WWLST	85	Severe three-domain involvement	3/85 (3.5)	2/85 (2.4)	-1.2
IVH3 entity non-neuro-WWLST	85	Selected CUS in CUS1–CUS2	47/85 (55.3)	14/85 (16.5)	-38.8
IVH3 entity non-neuro-WWLST	85	Selected CUS in CUS3–CUS4	38/85 (44.7)	71/85 (83.5)	+38.8
cPVL non-neuro-WWLST	65	Last available lower burden than maximal CUS	0/65 (0.0)	12/65 (18.5)	
cPVL non-neuro-WWLST	65	Severe-domain count, median [IQR]	1 [1–2]	1 [1–1]	
cPVL non-neuro-WWLST	65	Parenchymal lesion ≥1 cm (LP4)	53/65 (81.5)	51/65 (78.5)	-3.1
cPVL non-neuro-WWLST	65	Grade 3 IVH	11/65 (16.9)	6/65 (9.2)	-7.7
cPVL non-neuro-WWLST	65	VM≥2	15/65 (23.1)	14/65 (21.5)	-1.5
cPVL non-neuro-WWLST	65	≥2 severe domains	19/65 (29.2)	16/65 (24.6)	-4.6

Group	n	Variable	Maximal-burden CUS	Last available CUS	Difference (last – maximal, percentage points)
cPVL non-neuro-WWLST	65	Severe three-domain involvement	5/65 (7.7)	2/65 (3.1)	-4.6
cPVL non-neuro-WWLST	65	Selected CUS in CUS1–CUS2	8/65 (12.3)	3/65 (4.6)	-7.7
cPVL non-neuro-WWLST	65	Selected CUS in CUS3–CUS4	57/65 (87.7)	62/65 (95.4)	+7.7
All SBI survivors only	197	Last available lower burden than maximal CUS	0/197 (0.0)	91/197 (46.2)	
All SBI survivors only	197	Severe-domain count, median [IQR]	1 [1–2]	1 [0–1]	
All SBI survivors only	197	Parenchymal lesion ≥1 cm (LP4)	90/197 (45.7)	82/197 (41.6)	-4.1
All SBI survivors only	197	Grade 3 IVH	97/197 (49.2)	33/197 (16.8)	-32.5
All SBI survivors only	197	VM≥2	79/197 (40.1)	66/197 (33.5)	-6.6
All SBI survivors only	197	≥2 severe domains	84/197 (42.6)	47/197 (23.9)	-18.8
All SBI survivors only	197	Severe three-domain involvement	17/197 (8.6)	7/197 (3.6)	-5.1
All SBI survivors only	197	Selected CUS in CUS1–CUS2	69/197 (35.0)	2/197 (1.0)	-34.0
All SBI survivors only	197	Selected CUS in CUS3–CUS4	128/197 (65.0)	195/197 (99.0)	+34.0
All SBI Death not classified as neuro-WWLST only	46	Last available lower burden than maximal CUS	0/46 (0.0)	6/46 (13.0)	
All SBI Death not classified as neuro-WWLST only	46	Severe-domain count, median [IQR]	1 [1–2]	1 [1–2]	
All SBI Death not classified as neuro-WWLST only	46	Parenchymal lesion ≥1 cm (LP4)	18/46 (39.1)	19/46 (41.3)	+2.2
All SBI Death not classified as neuro-WWLST only	46	Grade 3 IVH	33/46 (71.7)	26/46 (56.5)	-15.2
All SBI Death not classified as neuro-WWLST only	46	VM≥2	10/46 (21.7)	10/46 (21.7)	+0.0
All SBI Death not classified as neuro-WWLST only	46	≥2 severe domains	16/46 (34.8)	15/46 (32.6)	-2.2
All SBI Death not classified as neuro-WWLST only	46	Severe three-domain involvement	3/46 (6.5)	2/46 (4.3)	-2.2
All SBI Death not classified as neuro-WWLST only	46	Selected CUS in CUS1–CUS2	35/46 (76.1)	28/46 (60.9)	-15.2
All SBI Death not classified as neuro-WWLST only	46	Selected CUS in CUS3–CUS4	11/46 (23.9)	18/46 (39.1)	+15.2

Note. Values compare last available CUS with maximal-burden CUS among infants not classified as neuro-WWLST deaths. Differences are shown as last available CUS minus maximal-burden CUS.

Supplementary Table S13. Fixed early-window CUS1–CUS2 imaging burden

Panel A. All SBI

Variable	Survivors	Death not classified as neuro-WWLST	Neuro-WWLST death	Neuro-WWLST vs survivors	Neuro-WWLST vs Death not classified as neuro-WWLST
All SBI					
n	197	46	43		
At least one evaluable early-window burden	197/197 (100.0)	46/46 (100.0)	43/43 (100.0)	Not calculated	Not calculated
Both early windows evaluable	194/197 (98.5)	33/46 (71.7)	30/43 (69.8)	Not calculated	Not calculated
Early-window maximal burden selected at CUS1	98/197 (49.7)	23/46 (50.0)	18/43 (41.9)	Not calculated	Not calculated
Early-window maximal burden selected at CUS2	99/197 (50.3)	23/46 (50.0)	25/43 (58.1)	Not calculated	Not calculated
Parenchymal lesion ≥1 cm by CUS1–CUS2	38/197 (19.3)	12/46 (26.1)	28/43 (65.1)	7.81 (3.80–16.05); p<0.001	5.29 (2.13–13.13); p<0.001
Grade 3 IVH by CUS1–CUS2	71/197 (36.0)	28/46 (60.9)	31/43 (72.1)	4.58 (2.22–9.49); p<0.001	1.66 (0.68–4.05); p=0.370
VM≥2 by CUS1–CUS2	35/197 (17.8)	7/46 (15.2)	19/43 (44.2)	3.66 (1.81–7.41); p<0.001	4.41 (1.61–12.05); p=0.005
≥2 severe domains by CUS1–CUS2	38/197 (19.3)	11/46 (23.9)	27/43 (62.8)	7.06 (3.46–14.40); p<0.001	5.37 (2.15–13.44); p<0.001
Severe three-domain involvement by CUS1–CUS2	6/197 (3.0)	1/46 (2.2)	13/43 (30.2)	13.79 (4.87–39.07); p<0.001	19.50 (2.42–156.99); p<0.001
Full maximal burden occurred after CUS2	128/197 (65.0)	11/46 (23.9)	10/43 (23.3)	0.16 (0.08–0.35); p<0.001	0.96 (0.36–2.57); p=1.000

Panel B. PVHI

Variable	Survivors	Death not classified as neuro-WWLST	Neuro-WWLST death	Neuro-WWLST vs survivors	Neuro-WWLST vs Death not classified as neuro-WWLST
PVHI					
n	75	18	24		
At least one evaluable early-window burden	75/75 (100.0)	18/18 (100.0)	24/24 (100.0)	Not calculated	Not calculated
Both early windows evaluable	75/75 (100.0)	10/18 (55.6)	15/24 (62.5)	Not calculated	Not calculated
Early-window maximal burden selected at CUS1	38/75 (50.7)	11/18 (61.1)	10/24 (41.7)	Not calculated	Not calculated
Early-window maximal burden selected at CUS2	37/75 (49.3)	7/18 (38.9)	14/24 (58.3)	Not calculated	Not calculated
Parenchymal lesion ≥1 cm by CUS1–CUS2	27/75 (36.0)	9/18 (50.0)	21/24 (87.5)	12.44 (3.40–45.59); p<0.001	7.00 (1.53–32.08); p=0.014
Grade 3 IVH by CUS1–CUS2	24/75 (32.0)	7/18 (38.9)	20/24 (83.3)	10.62 (3.27–34.51); p<0.001	7.86 (1.88–32.90); p=0.004
VM≥2 by CUS1–CUS2	12/75 (16.0)	1/18 (5.6)	13/24 (54.2)	6.20 (2.25–17.08); p<0.001	20.09 (2.29–176.10); p<0.001
≥2 severe domains by CUS1–CUS2	18/75 (24.0)	3/18 (16.7)	20/24 (83.3)	15.83 (4.78–52.42); p<0.001	25.00 (4.85–128.86); p<0.001

Variable	Survivors	Death not classified as neuro-WWLST	Neuro-WWLST death	Neuro-WWLST vs survivors	Neuro-WWLST vs Death not classified as neuro-WWLST
Severe three-domain involvement by CUS1–CUS2	5/75 (6.7)	0/18 (0.0)	11/24 (45.8)	11.85 (3.53–39.79); p<0.001	∞ (1.70–582.70); p<0.001
Full maximal burden occurred after CUS2	40/75 (53.3)	4/18 (22.2)	2/24 (8.3)	0.08 (0.02–0.36); p<0.001	0.32 (0.05–1.97); p=0.375

Panel C. IVH3 entity

Variable	Survivors	Death not classified as neuro-WWLST	Neuro-WWLST death	Neuro-WWLST vs survivors	Neuro-WWLST vs Death not classified as neuro-WWLST
IVH3 entity					
n	66	19	9		
At least one evaluable early-window burden	65/66 (98.5)	19/19 (100.0)	9/9 (100.0)	Not calculated	Not calculated
Both early windows evaluable	66/66 (100.0)	15/19 (78.9)	6/9 (66.7)	Not calculated	Not calculated
Early-window maximal burden selected at CUS1	22/66 (33.3)	6/19 (31.6)	3/9 (33.3)	Not calculated	Not calculated
Early-window maximal burden selected at CUS2	44/66 (66.7)	13/19 (68.4)	6/9 (66.7)	Not calculated	Not calculated
Parenchymal lesion ≥1 cm by CUS1–CUS2	1/66 (1.5)	2/19 (10.5)	2/9 (22.2)	18.57 (1.49–231.73); p=0.036	2.43 (0.28–20.82); p=0.574
Grade 3 IVH by CUS1–CUS2	42/66 (63.6)	18/19 (94.7)	9/9 (100.0)	∞ (0.61–196.49); p=0.050	∞ (0.06–41.56); p=1.000
VM≥2 by CUS1–CUS2	19/66 (28.8)	6/19 (31.6)	4/9 (44.4)	1.98 (0.48–8.18); p=0.443	1.73 (0.34–8.87); p=0.677
≥2 severe domains by CUS1–CUS2	16/66 (24.2)	7/19 (36.8)	5/9 (55.6)	3.91 (0.93–16.33); p=0.106	2.14 (0.43–10.74); p=0.432
Severe three-domain involvement by CUS1–CUS2	1/66 (1.5)	1/19 (5.3)	1/9 (11.1)	8.12 (0.46–142.94); p=0.227	2.25 (0.12–40.66); p=1.000
Full maximal burden occurred after CUS2	37/66 (56.1)	1/19 (5.3)	2/9 (22.2)	0.22 (0.04–1.16); p=0.079	5.14 (0.40–66.15); p=0.234

Panel D. cPVL

Variable	Survivors	Death not classified as neuro-WWLST	Neuro-WWLST death	Neuro-WWLST vs survivors	Neuro-WWLST vs Death not classified as neuro-WWLST
cPVL					
n	56	9	10		
At least one evaluable early-window burden	56/56 (100.0)	9/9 (100.0)	10/10 (100.0)	Not calculated	Not calculated
Both early windows evaluable	54/56 (96.4)	8/9 (88.9)	9/10 (90.0)	Not calculated	Not calculated
Early-window maximal burden selected at CUS1	38/56 (67.9)	6/9 (66.7)	5/10 (50.0)	Not calculated	Not calculated
Early-window maximal burden selected at CUS2	18/56 (32.1)	3/9 (33.3)	5/10 (50.0)	Not calculated	Not calculated

Variable	Survivors	Death not classified as neuro-WWLST	Neuro-WWLST death	Neuro-WWLST vs survivors	Neuro-WWLST vs Death not classified as neuro-WWLST
Parenchymal lesion ≥1 cm by CUS1–CUS2	10/56 (17.9)	1/9 (11.1)	5/10 (50.0)	4.60 (1.12–18.95); p=0.040	8.00 (0.71–90.00); p=0.141
Grade 3 IVH by CUS1–CUS2	5/56 (8.9)	3/9 (33.3)	2/10 (20.0)	2.55 (0.42–15.45); p=0.285	0.50 (0.06–4.00); p=0.628
VM≥2 by CUS1–CUS2	4/56 (7.1)	0/9 (0.0)	2/10 (20.0)	3.25 (0.51–20.74); p=0.222	∞ (0.23–133.61); p=0.474
≥2 severe domains by CUS1–CUS2	4/56 (7.1)	1/9 (11.1)	2/10 (20.0)	3.25 (0.51–20.74); p=0.222	2.00 (0.15–26.74); p=1.000
Severe three-domain involvement by CUS1–CUS2	0/56 (0.0)	0/9 (0.0)	1/10 (10.0)	∞ (0.68–471.16); p=0.152	∞ (0.11–83.36); p=1.000
Full maximal burden occurred after CUS2	51/56 (91.1)	6/9 (66.7)	6/10 (60.0)	0.15 (0.03–0.70); p=0.024	0.75 (0.11–4.90); p=1.000

Note. The fixed early-window analysis used CUS1 and CUS2 only. This sensitivity analysis describes burden documented within fixed early windows and does not replace the full serial maximal-burden CUS analysis.

Supplementary Table S14. Restricted adjusted and sparse-data sensitivity models

Panel A. Restricted adjusted and sparse-data sensitivity models for neuro-WWLST death

Population	Exposure	n/events	Exposure among neuro-WWLST	Exposure among non-neuro-WWLST	Unadjusted OR (95% CI); Fisher p	Restricted adjusted OR (95% CI); p	Notes
All SBI	Severe three-domain involvement at maximal-burden CUS	286/43	16/43 (37.2)	20/243 (8.2)	6.61 (3.06–14.26); p<0.001	7.18 (3.00–17.16); p<0.001	Adjusted for GA, birth epoch and SBI entity
All SBI	≥2 severe domains at maximal-burden CUS	286/43	30/43 (69.8)	100/243 (41.2)	3.30 (1.64–6.64); p<0.001	3.69 (1.71–7.96); p<0.001	Adjusted for GA, birth epoch and SBI entity
All SBI	Parenchymal lesion ≥1 cm at maximal-burden CUS	286/43	35/43 (81.4)	108/243 (44.4)	5.47 (2.44–12.28); p<0.001	8.83 (2.92–26.68); p<0.001	Adjusted for GA, birth epoch and SBI entity
All SBI	Grade 3 IVH at maximal-burden CUS	286/43	31/43 (72.1)	130/243 (53.5)	2.25 (1.10–4.58); p=0.030	4.59 (1.91–11.00); p<0.001	Adjusted for GA, birth epoch and SBI entity
All SBI	VM≥2 at maximal-burden CUS	286/43	22/43 (51.2)	89/243 (36.6)	1.81 (0.94–3.48); p=0.089	2.24 (1.04–4.81); p=0.039	Adjusted for GA, birth epoch and SBI entity
PVHI	Severe three-domain involvement at maximal-burden CUS	117/24	12/24 (50.0)	12/93 (12.9)	6.75 (2.47–18.42); p<0.001	10.95 (3.10–38.67); p<0.001	Entity-specific sparse-data sensitivity
PVHI	Coexisting grade 3 IVH at maximal-burden CUS	117/24	20/24 (83.3)	35/93 (37.6)	8.29 (2.62–26.24); p<0.001	8.09 (2.47–26.49); p<0.001	Entity-specific sparse-data sensitivity
PVHI	VM≥2 at maximal-burden CUS	117/24	14/24 (58.3)	26/93 (28.0)	3.61 (1.42–9.14); p=0.008	3.88 (1.37–11.00); p=0.011	Entity-specific sparse-data sensitivity
PVHI	≥2 severe domains at maximal-burden CUS	117/24	21/24 (87.5)	32/93 (34.4)	13.34 (3.70–48.14); p<0.001	12.58 (3.39–46.71); p<0.001	Entity-specific sparse-data sensitivity
IVH3 entity	Parenchymal lesion ≥1 cm at maximal-burden CUS	94/9	3/9 (33.3)	5/85 (5.9)	8.00 (1.53–41.84); p=0.027	Not fitted (sparse entity-specific events)	Entity-specific sparse-data sensitivity
IVH3 entity	VM≥2 at maximal-burden CUS	94/9	5/9 (55.6)	48/85 (56.5)	0.96 (0.24–3.84); p=1.000	Not fitted (sparse entity-specific events)	Entity-specific sparse-data sensitivity
IVH3 entity	Severe three-domain involvement at maximal-burden CUS	94/9	2/9 (22.2)	3/85 (3.5)	7.81 (1.11–54.81); p=0.070	Not fitted (sparse entity-specific events)	Entity-specific sparse-data sensitivity
cPVL	Severe three-domain involvement at maximal-burden CUS	75/10	2/10 (20.0)	5/65 (7.7)	3.00 (0.50–18.12); p=0.233	5.23 (0.56–49.14); p=0.148	Entity-specific sparse-data sensitivity
cPVL	VM≥2 at maximal-burden CUS	75/10	3/10 (30.0)	15/65 (23.1)	1.43 (0.33–6.22); p=0.695	1.11 (0.21–5.89); p=0.907	Entity-specific sparse-data sensitivity
cPVL	Grade 3 IVH at maximal-burden CUS	75/10	2/10 (20.0)	11/65 (16.9)	1.23 (0.23–6.58); p=1.000	1.95 (0.28–13.83); p=0.502	Entity-specific sparse-data sensitivity
cPVL	Parenchymal lesion ≥1 cm at maximal-burden CUS	75/10	10/10 (100.0)	53/65 (81.5)	∞ (0.27–89.45); p=0.349	Not estimable because all neuro-WWLST deaths had parenchymal lesion ≥1 cm.	Entity-specific sparse-data sensitivity

Panel B. Sensitivity models replacing gestational age with birth weight

Outcome	N analyzed	Events	Exposure	Adjustment set	Adjusted OR (95% CI)	p value
Neuro-WWLST death within SBI	286	43	Severe three-domain involvement at common maximal-burden CUS	Birth weight, birth epoch, SBI entity	8.37 (3.44–20.35)	<0.001

Outcome	N analyzed	Events	Exposure	Adjustment set	Adjusted OR (95% CI)	p value
Cerebral palsy among SBI survivors	176	87	Severe three-domain involvement	Birth weight, birth epoch, SBI entity	5.02 (1.46–17.25)	0.010
Clinically classified school-age cognitive sequelae among SBI survivors	155	50	Severe three-domain involvement	Birth weight, birth epoch, SBI entity	3.52 (1.12–11.02)	0.031

Note. Models were interpreted as sensitivity analyses and were not intended for prediction or causal inference. Gestational age and birth weight were not entered together because of collinearity and sparse event counts.

Supplementary Table S15. PVHI: gestational age and neuro-WWLST by coexisting grade 3 IVH, overall and by debut window

Scope	All	CUS1–CUS2	CUS3–CUS4
PVHI with coexisting grade 3 IVH, n	56	50	6
PVHI with coexisting grade 3 IVH, GA, median [IQR]	26 [25–28]	26 [25–28]	25.5 [24.2–26]
PVHI with coexisting grade 3 IVH, neuro-WWLST	20/56 (35.7%)	19/50 (38.0%)	1/6 (16.7%)
PVHI with maximal IVH <3, n	61	50	11
PVHI with maximal IVH <3, GA, median [IQR]	27 [25–30]	26 [25–29]	29 [27–30]
PVHI with maximal IVH <3, neuro-WWLST	4/61 (6.6%)	4/50 (8.0%)	0/11 (0.0%)
OR (Wald 95% CI)	7.92 (2.50–25.05)	7.05 (2.19–22.72)	6.27 (0.22–180.24)†
Fisher p	<0.001	<0.001	0.353
Mann–Whitney p (GA)	0.137	0.728	0.011

Note. PVHI with coexisting grade 3 IVH denotes PVHI with maximal coexisting IVH grade 3; PVHI with maximal IVH <3 denotes PVHI with maximal coexisting IVH <3. Columns stratify by PVHI debut window. † Haldane–Anscombe correction was applied because of a zero cell.

Supplementary Table S16. IVH3 entity: gestational age and neuro-WWLST by additional parenchymal injury versus isolated IVH3

Scope	All	CUS1–CUS2
IVH3 entity with additional parenchymal injury, n	8	8
IVH3 entity with additional parenchymal injury, GA, median [IQR]	27 [26–28.8]	27 [26–28.8]
IVH3 entity with additional parenchymal injury, neuro-WWLST	4/8 (50.0%)	4/8 (50.0%)
Isolated IVH3, n	86	61
Isolated IVH3, GA, median [IQR]	27 [25–28]	27 [25–28]
Isolated IVH3, neuro-WWLST	5/86 (5.8%)	5/61 (8.2%)
OR (Wald 95% CI)	16.20 (3.10–84.71)	11.20 (2.13–58.94)
Fisher p	0.002	0.008
Mann–Whitney p (GA)	0.498	0.562

Note. Additional parenchymal injury included arterial infarction/hemorrhage (lobar or cerebellar) and mixed focal parenchymal lesions not fulfilling PVHI or cPVL criteria.

Supplementary Table S17. cPVL: gestational age and neuro-WWLST by coexisting grade 3 IVH, overall and by debut window

Scope	All	CUS1–CUS2	CUS3–CUS4
cPVL with coexisting grade 3 IVH, n	16	8	8
cPVL with coexisting grade 3 IVH, GA, median [IQR]	26 [26–28]	27 [26–28]	26 [26–27.8]
cPVL with coexisting grade 3 IVH, neuro-WWLST	2/16 (12.5%)	1/8 (12.5%)	1/8 (12.5%)
cPVL with maximal IVH <3, n	59	18	41
cPVL with maximal IVH <3, GA, median [IQR]	28 [27–30]	29 [27.2–30]	28 [27–30]
cPVL with maximal IVH <3, neuro-WWLST	8/59 (13.6%)	4/18 (22.2%)	4/41 (9.8%)
OR (Wald 95% CI)	0.91 (0.17–4.78)	0.50 (0.05–5.36)	1.32 (0.13–13.66)
Fisher p	1.000	1.000	1.000
Mann–Whitney p (GA)	0.114	0.207	0.263

Note. cPVL with coexisting grade 3 IVH denotes cPVL with maximal coexisting IVH grade 3; cPVL with maximal IVH <3 denotes cPVL with maximal coexisting IVH <3. Columns stratify by cPVL debut window.

Supplementary Table S18. Neurodevelopmental follow-up availability among SBI survivors

Entity	Survivors	CP follow-up available	School-age cognitive follow-up available
All SBI survivors	197	176/197 (89.3%)	155/197 (78.7%)
PVHI	75	69/75 (92.0%)	64/75 (85.3%)
IVH3 entity	66	57/66 (86.4%)	50/66 (75.8%)
cPVL	56	50/56 (89.3%)	41/56 (73.2%)

Note. Values are n/N (%). Analyses are restricted to survivors within the three SBI entities. CP was assessed at approximately 2 years' corrected age. School-age cognitive outcome was classified from longitudinal follow-up records, usually around the 7-year assessment window.

Supplementary Table S19. Cerebral palsy and GMFCS among survivors by SBI entity and severe-domain count

Entity	No. severe imaging domains	Survivors, n	Cerebral palsy, n/N (%)	GMFCS I	GMFCS II	GMFCS III	GMFCS IV	GMFCS V
All SBI survivors	0	32	12/31 (38.7%)	8/12	1/12	3/12	0/12	0/12
All SBI survivors	1	84	42/70 (60.0%)	15/42	7/42	13/42	5/42	2/42
All SBI survivors	2	63	20/58 (34.5%)	10/20	4/20	2/20	1/20	3/20
All SBI survivors	3	18	13/17 (76.5%)	4/13	5/13	2/13	1/13	1/13
PVHI	0	23	7/23 (30.4%)	6/7	1/7	0/7	0/7	0/7
PVHI	1	23	10/21 (47.6%)	8/10	1/10	1/10	0/10	0/10
PVHI	2	18	5/15 (33.3%)	4/5	1/5	0/5	0/5	0/5
PVHI	3	11	7/10 (70.0%)	3/7	4/7	0/7	0/7	0/7
IVH3 entity	0	0	—	—	—	—	—	—
IVH3 entity	1	30	6/21 (28.6%)	4/6	1/6	0/6	1/6	0/6
IVH3 entity	2	33	7/33 (21.2%)	3/7	2/7	1/7	0/7	1/7
IVH3 entity	3	3	3/3 (100.0%)	0/3	0/3	2/3	0/3	1/3
cPVL	0	9	5/8 (62.5%)	2/5	0/5	3/5	0/5	0/5
cPVL	1	31	26/28 (92.9%)	3/26	5/26	12/26	4/26	2/26
cPVL	2	12	8/10 (80.0%)	3/8	1/8	1/8	1/8	2/8
cPVL	3	4	3/4 (75.0%)	1/3	1/3	0/3	1/3	0/3

Note. Analyses are restricted to survivors with available CP follow-up data. Severe imaging domains were parenchymal lesion ≥1 cm, grade 3 IVH and VM≥2, assessed at maximal-burden CUS. GMFCS grades are shown among CP cases with available functional grading.

Supplementary Table S20. Cerebral palsy motor phenotype among survivors with CP by SBI entity and severe-domain count

Entity	No. severe imaging domains	CP cases with phenotype available	Unilateral/hemiparetic CP	Diplegic/paraparetic CP	Triparetic/tetraparetic CP	Other/mixed CP phenotype
All SBI survivors	0	12	8/12 (66.7%)	3/12 (25.0%)	1/12 (8.3%)	0/12 (0.0%)
All SBI survivors	1	42	11/42 (26.2%)	20/42 (47.6%)	11/42 (26.2%)	0/42 (0.0%)
All SBI survivors	2	20	7/20 (35.0%)	9/20 (45.0%)	2/20 (10.0%)	2/20 (10.0%)
All SBI survivors	3	13	9/13 (69.2%)	1/13 (7.7%)	3/13 (23.1%)	0/13 (0.0%)
PVHI	0	7	7/7 (100.0%)	0/7 (0.0%)	0/7 (0.0%)	0/7 (0.0%)
PVHI	1	10	10/10 (100.0%)	0/10 (0.0%)	0/10 (0.0%)	0/10 (0.0%)
PVHI	2	5	5/5 (100.0%)	0/5 (0.0%)	0/5 (0.0%)	0/5 (0.0%)
PVHI	3	7	7/7 (100.0%)	0/7 (0.0%)	0/7 (0.0%)	0/7 (0.0%)
IVH3 entity	0	—	—	—	—	—
IVH3 entity	1	6	0/6 (0.0%)	6/6 (100.0%)	0/6 (0.0%)	0/6 (0.0%)
IVH3 entity	2	7	0/7 (0.0%)	5/7 (71.4%)	0/7 (0.0%)	2/7 (28.6%)
IVH3 entity	3	3	0/3 (0.0%)	1/3 (33.3%)	2/3 (66.7%)	0/3 (0.0%)
cPVL	0	5	1/5 (20.0%)	3/5 (60.0%)	1/5 (20.0%)	0/5 (0.0%)
cPVL	1	26	1/26 (3.8%)	14/26 (53.8%)	11/26 (42.3%)	0/26 (0.0%)
cPVL	2	8	2/8 (25.0%)	4/8 (50.0%)	2/8 (25.0%)	0/8 (0.0%)
cPVL	3	3	2/3 (66.7%)	0/3 (0.0%)	1/3 (33.3%)	0/3 (0.0%)

Note. CP phenotype was classified from the registry cerebral palsy phenotype variable. Phenotype-specific analyses were descriptive only because strata were small.

Supplementary Table S21. Clinically classified school-age cognitive sequelae among survivors by SBI entity and severe-domain count

Entity	No. severe imaging domains	Survivors, n	School-age cognitive sequelae, n/N (%)	Mild School-age cognitive sequelae	Moderate School-age cognitive sequelae	Severe School-age cognitive sequelae
All SBI survivors	0	32	6/26 (23.1%)	5/26	1/26	0/26
All SBI survivors	1	84	22/63 (34.9%)	16/63	6/63	0/63
All SBI survivors	2	63	13/49 (26.5%)	9/49	4/49	0/49
All SBI survivors	3	18	9/17 (52.9%)	7/17	0/17	2/17
PVHI	0	23	2/20 (10.0%)	2/20	0/20	0/20
PVHI	1	23	5/21 (23.8%)	4/21	1/21	0/21
PVHI	2	18	2/13 (15.4%)	0/13	2/13	0/13
PVHI	3	11	4/10 (40.0%)	4/10	0/10	0/10
IVH3 entity	0	0	—	—	—	—
IVH3 entity	1	30	4/19 (21.1%)	3/19	1/19	0/19
IVH3 entity	2	33	7/28 (25.0%)	5/28	2/28	0/28
IVH3 entity	3	3	3/3 (100.0%)	3/3	0/3	0/3
cPVL	0	9	4/6 (66.7%)	3/6	1/6	0/6
cPVL	1	31	13/23 (56.5%)	9/23	4/23	0/23
cPVL	2	12	4/8 (50.0%)	4/8	0/8	0/8
cPVL	3	4	2/4 (50.0%)	0/4	0/4	2/4

Note. Analyses are restricted to survivors with available school-age follow-up data. Cognitive sequelae were clinically classified as absent, mild, moderate or severe from longitudinal follow-up information.

Supplementary Table S22. Exploratory adjusted associations between severe three-domain involvement and survivor outcomes

Outcome	N analyzed	Events	Severe three-domain involvement	Fewer than three severe domains	Unadjusted p	Adjusted OR (95% CI)	Adjusted p
Cerebral palsy	176	87	13/17 (76.5%)	74/159 (46.5%)	0.022	4.99 (1.45–17.17)	0.011
School-age cognitive sequelae	155	50	9/17 (52.9%)	41/138 (29.7%)	0.096	3.72 (1.19–11.65)	0.024

Note. Logistic regression models included severe involvement across all three imaging domains as the exposure and were adjusted for gestational age, birth epoch and SBI entity. Analyses were exploratory and were not intended for prediction.

Supplementary Table S23. Scheduled CUS window availability across SBI entities

**Window**	**PVHI (n=117)**	**IVH3 entity (n=94)**	**cPVL (n=75)**
CUS1 (≤48 h)	117/117 (100.0%)	94/94 (100.0%)	75/75 (100.0%)
CUS2 (~day 7)	100/117 (85.5%)	86/94 (91.5%)	71/75 (94.7%)
CUS3 (~day 28)	80/117 (68.4%)	72/94 (76.6%)	68/75 (90.7%)
CUS4 (TEA/discharge)	76/117 (65.0%)	70/94 (74.5%)	61/75 (81.3%)

Note. Values are n/N (%), where N is the entity total. Scheduled window availability indicates that at least one of the three imaging-domain grades (LP, IVH or VM) was recorded in that scheduled CUS window.

Supplementary Table S24. Complete three-domain availability across SBI entities

**Window**	**PVHI (n=117)**	**IVH3 entity (n=94)**	**cPVL (n=75)**
CUS1 (≤48 h)	117/117 (100.0%)	91/94 (96.8%)	74/75 (98.7%)
CUS2 (~day 7)	100/117 (85.5%)	85/94 (90.4%)	71/75 (94.7%)
CUS3 (~day 28)	80/117 (68.4%)	72/94 (76.6%)	67/75 (89.3%)
CUS4 (TEA/discharge)	76/117 (65.0%)	70/94 (74.5%)	61/75 (81.3%)

Note. Values are n/N (%), where N is the entity total. Complete three-domain availability indicates that LP, IVH and VM grades were all available in that scheduled CUS window.

Supplementary Table S25. Algorithm-resolution profile for maximal-burden CUS selection in PVHI without neuro-WWLST

**Hierarchy step**	**Definition**	**PVHI without neuro-WWLST (n=93)**
Step 1	Maximise number of high-burden domains present (parenchymal lesion ≥1 cm, grade 3 IVH, VM≥2)	31/93 (33.3%)
Step 2	If tied, maximise joint severity sum (LP+IVH+VM)	15/93 (16.1%)
Step 3	If still tied, select earliest CUS window	47/93 (50.5%)
Sensitivity check	Alternative tie-break (LP→VM→IVH→earliest): reference CUS window changed	7/93 (7.5%)

Note. PVHI without neuro-WWLST includes PVHI survivors and PVHI deaths without neuro-WWLST (n=93). Percentages in steps 1–3 indicate the proportion of infants whose final maximal-burden CUS selection was resolved at that hierarchy step.

Supplementary Table S26. Sensitivity to alternative tie-breaking rules for maximal-burden CUS selection in PVHI without neuro-WWLST

Metric	Baseline (sum→earliest)	Alternative (LP→VM→IVH, then earliest)	Difference
Reference CUS window changed	—	7/93 (7.5%)	+7.5 percentage points
Severe involvement across all three domains at selected reference CUS	12/93 (12.9%)	12/93 (12.9%)	+0.0 pp
Parenchymal lesion ≥1 cm at selected reference CUS	50/93 (53.8%)	51/93 (54.8%)	+1.1 pp
Grade 3 IVH at selected reference CUS	35/93 (37.6%)	33/93 (35.5%)	−2.2 pp
VM≥2 at selected reference CUS	26/93 (28.0%)	27/93 (29.0%)	+1.1 pp

Note. The baseline maximal-burden CUS selection used the prespecified hierarchy. The alternative rule prioritized LP, then VM, then IVH, before the earliest-window rule after step 1. Severe three-domain involvement denotes concurrent parenchymal lesion ≥1 cm, grade 3 IVH and VM≥2.

Abbreviations: BW, birth weight; CI, confidence interval; CP, cerebral palsy; cPVL, cystic periventricular leukomalacia; CUS, cranial ultrasound; GA, gestational age; GMFCS, Gross Motor Function Classification System; IVH, intraventricular hemorrhage; LP, parenchymal lesion; OR, odds ratio; PVHI, periventricular hemorrhagic infarction; SBI, severe brain injury; VM, ventriculomegaly; VP, ventriculoperitoneal; WWLST, withdrawal, withholding or non-escalation of life-sustaining treatment.

Supplementary Table S27. STROBE checklist for cohort studies

Section	Item No.	Recommendation	Page No.	Relevant text from manuscript
**Title and abstract**	1a	Indicate the study's design with a commonly used term in the title or the abstract	Main p. 1–2	The title identifies the article as a "Single-Center Cohort Study." The abstract Methods identifies a retrospective single-center cohort of 2,841 very preterm infants from 1991–2020 with complete CUS within 48 h after birth.
Title and abstract	1b	Provide in the abstract an informative and balanced summary of what was done and what was found	Main p. 1–2	The structured abstract summarizes the background/objectives, methods, results and conclusions, including the cohort denominator, four-window/three-domain CUS framework, mutually exclusive SBI entities, deaths following documented neuro-WWLST, survivor outcomes, and cautious descriptive conclusions.
**Introduction**	2	Explain the scientific background and rationale for the investigation being reported	Main p. 2–3	The Introduction explains that SBI in very preterm infants includes heterogeneous lesion types with different timing, imaging burden, mortality and neurodevelopmental outcomes, and that serial CUS may describe documented classifiability, multidomain burden and outcome-proximal imaging context.
Introduction	3	State specific objectives, including any prespecified hypotheses	Main p. 4	The objectives are stated explicitly: characterize PVHI, the IVH3 entity and cPVL by documented timing and three-domain burden; compare in-hospital outcome groups using a common maximal-burden CUS definition; describe last-window CUS among deaths following documented neuro-WWLST; and exploratorily describe survivor outcomes.

Section	Item No.	Recommendation	Page No.	Relevant text from manuscript
**Methods**	4	Present key elements of study design early in the paper	Main p. 4	The Methods open with: "This retrospective cohort study used a prospectively maintained single-center tertiary NICU registry from 1991 to 2020."
Methods	5	Describe the setting, locations, and relevant dates, including periods of recruitment, exposure, follow-up, and data collection	Main p. 4; Supplement p. 1–2 (Methods S1–S2)	The setting is a single-center tertiary NICU registry from 1991–2020. In-hospital outcomes were assessed until discharge or death; CP was assessed at approximately 2 years' corrected age and school-age cognitive outcome around the routine 7-year follow-up window when available.
Methods	6a	Cohort study—Give eligibility criteria, sources and methods of selection of participants. Describe methods of follow-up	Main p. 4; Figure 1 p. 7; Supplement p. 1 and p. 13 (S1; Table S1)	Eligible infants were born at <32 weeks' GA and/or had BW ≤1500 g. Imaging-based analyses were restricted to infants with complete recorded CUS1 within 48 h. Supplementary Methods S1 and Supplementary Table S1 describe the CUS1-imaged denominator and excluded infants without complete CUS1.
Methods	6b	Cohort study—For matched studies, give matching criteria and number of exposed and unexposed	Not applicable	Not applicable. This was not a matched cohort study.
Methods	7	Clearly define all outcomes, exposures, predictors, potential confounders, and effect modifiers. Give diagnostic criteria, if applicable	Main p. 4–6; Supplement p. 2–11 (Methods S2–S7)	Outcomes were survival to discharge, death not classified as neuro-WWLST, death following documented neuro-WWLST, CP and clinically classified school-age cognitive sequelae. Imaging features included SBI entity, CUS window, parenchymal lesion, IVH, VM, severe imaging-domain count and severe three-domain involvement. GA, BW, birth epoch and SBI entity are described as covariates in sensitivity models.

Section	Item No.	Recommendation	Page No.	Relevant text from manuscript
Methods	8	For each variable of interest, give sources of data and methods of assessment. Describe comparability of assessment methods if there is more than one group	Main p. 4–5; Supplement p. 1–11 (Methods S1–S7)	Clinical, imaging and outcome variables were obtained from the prospectively maintained NICU registry, contemporaneous radiology reports, clinical/radiology records and longitudinal follow-up records. CUS surveillance windows, reporting source, grading, entity definitions, MRI availability and neurodevelopmental follow-up methods are detailed in the Supplementary Methods.
Methods	9	Describe any efforts to address potential sources of bias	Main p. 5–6 and p. 24–25; Supplement p. 6–9 (Methods S6)	Potential bias was addressed by prespecified CUS windows, mutually exclusive SBI definitions, common maximal-burden CUS as the primary cross-outcome comparator, explicit denominators, CUS-window availability reporting, sensitivity analyses, restricted adjusted models, complete-case reporting and expanded limitations on secular trends, MRI availability and follow-up heterogeneity.
Methods	10	Explain how the study size was arrived at	Supplement p. 1 (Methods S1)	No formal sample size calculation was performed; all eligible infants recorded in the prospectively maintained registry during 1991–2020 were considered. The CUS1-imaged analytic denominator was 2,841 infants.

Section	Item No.	Recommendation	Page No.	Relevant text from manuscript
Methods	11	Explain how quantitative variables were handled in the analyses. If applicable, describe which groupings were chosen and why	Main p. 5–6; Supplement p. 3–4 and p. 7–11 (Methods S3, S6–S7)	Continuous variables were summarized as median [IQR] and categorical variables as n/N (%). Prespecified groupings included CUS windows, GA groups, birth epochs, SBI entities, LP/IVH/VM grades, severe imaging domains, severe three-domain involvement, CP functional severity and cognitive outcome categories.
Methods	12a	Describe all statistical methods, including those used to control for confounding	Main p. 5–6; Supplement p. 6–8 (Methods S6)	The manuscript describes Mann–Whitney U tests, Fisher's exact tests, Hodges–Lehmann shifts, unadjusted odds ratios with 95% CIs, bootstrap CIs, Haldane–Anscombe correction for zero cells and exploratory logistic regression adjusted for GA, birth epoch and SBI entity. Sensitivity models replacing GA with BW are described.
Methods	12b	Describe any methods used to examine subgroups and interactions	Main p. 12–20; Supplement p. 6–11 (Methods S6–S7)	Subgroup and exploratory analyses were performed by SBI entity, in-hospital outcome group, severe-domain count, delayed timing, within-entity modifiers and survivor outcomes. Entity-specific panels and survivor outcome models are described as exploratory/descriptive.
Methods	12c	Explain how missing data were addressed	Main p. 5–6; Supplement p. 8–9 (Methods S6); Supplement Tables S1, S10, S18, S23–S24	Missing data were addressed using explicit denominators and complete-case analyses. CUS1-imaged exclusions are reported in Table S1; scheduled CUS-window availability in Table S10; additional CUS-window and complete three-domain availability in Tables S23–S24; and neurodevelopmental follow-up availability in Table S18. No imputation was performed.

Section	Item No.	Recommendation	Page No.	Relevant text from manuscript
Methods	12d	Cohort study—If applicable, explain how loss to follow-up was addressed	Main p. 19–20; Supplement p. 9–11 (Methods S7); Supplement Table S18	Neurodevelopmental analyses were restricted to survivors with available follow-up data for each outcome. CP and cognitive follow-up availability are summarized in the manuscript and in Supplementary Table S18.
Methods	12e	Describe any sensitivity analyses	Main p. 14–15; Supplement p. 6–9 (Methods S6); Supplement Tables S11–S14 and S25–S26	Sensitivity and robustness analyses included concordance between last-window and maximal-burden CUS among deaths following documented neuro-WWLST, last available CUS versus maximal-burden CUS among non-neuro-WWLST infants, fixed early-window CUS1–CUS2 analyses, restricted adjusted/sparse-data models and algorithm-specific/tie-breaking robustness checks.
**Results**	13a	Report numbers of individuals at each stage of study	Main p. 6–7 and Figure 1 p. 7; Supplement Table S1 p. 13	The manuscript reports 3,081 eligible very preterm infants, 2,841 in the CUS1-imaged denominator, 286 infants with SBI, 197 SBI survivors, 89 SBI deaths and 43 SBI deaths following documented neuro-WWLST. Follow-up denominators for CP and cognitive outcomes are reported in the survivor outcome section.
Results	13b	Give reasons for non-participation at each stage	Main p. 6–7; Supplement p. 1 and p. 13 (Methods S1; Table S1)	Infants without complete CUS1 were excluded from imaging-based analyses; reasons included deaths before NICU admission, early death, transfer or logistical reasons before complete CUS1 could be recorded.

Section	Item No.	Recommendation	Page No.	Relevant text from manuscript
Results	13c	Consider use of a flow diagram	Main Figure 1 p. 7	Figure 1 provides the flow diagram from source cohort to CUS1-imaged denominator, in-hospital outcomes, SBI cases, SBI entities and deaths following documented neuro-WWLST with SBI.
Results	14a	Give characteristics of study participants and information on exposures and potential confounders	Main Table 1 p. 11–12; Supplement Tables S1–S8 p. 13–21	Participant characteristics are reported by SBI entity, gestational age group, birth decade and outcome. Table 1 includes GA, BW, birth epoch and in-hospital outcomes across SBI entities.
Results	14b	Indicate number of participants with missing data for each variable of interest	Main p. 19–20; Supplement Tables S1, S10, S18, S23–S24	The manuscript and supplement provide explicit denominators for CUS1 availability, CUS-window availability, three-domain completeness and neurodevelopmental follow-up availability.
Results	14c	Cohort study—Summarize follow-up time	Main p. 4–5 and p. 19; Supplement p. 9–11 (Methods S7)	In-hospital outcomes were assessed until discharge or death. CP was assessed at approximately 2 years' corrected age. School-age cognitive sequelae were usually classified around the preschool-to-school transition or the routine 7-year assessment window when available.
Results	15	Cohort study—Report numbers of outcome events or summary measures over time	Main p. 6–20; Tables 1–4; Figures 1–5; Supplement Tables S5–S22	Outcome events are reported for survival, death not classified as neuro-WWLST, death following documented neuro-WWLST, entity-specific imaging patterns, last-window CUS, CP, non-ambulatory CP, motor phenotype, school-age cognitive sequelae and exploratory adjusted survivor outcome associations.

Section	Item No.	Recommendation	Page No.	Relevant text from manuscript
Results	16a	Give unadjusted estimates and, if applicable, confounder-adjusted estimates and their precision. Make clear which confounders were adjusted for and why	Main p. 12–20; Figure 4 p. 15–16; Supplement Tables S9, S14 and S22	Unadjusted estimates are reported in Table 2, Figure 4 and Supplementary Table S9. Restricted adjusted/sparse-data sensitivity models are reported in Supplementary Table S14. Exploratory adjusted survivor outcome models for CP and cognitive sequelae are reported in Supplementary Table S22; models adjusted for GA, birth epoch and SBI entity, with BW sensitivity models.
Results	16b	Report category boundaries when continuous variables were categorized	Main p. 4–5; Supplement p. 3–4 and p. 9–11 (Methods S3, S7)	Category boundaries are specified for GA groups, CUS windows, LP grades, IVH grades, VM grades, parenchymal lesion ≥1 cm, VM≥2, severe imaging-domain count, SBI entities, GMFCS categories and cognitive outcome categories.
Results	16c	If relevant, consider translating estimates of relative risk into absolute risk for a meaningful time period	Main p. 6–20; Tables 1–4; Figures 1–5; Supplement Tables S1–S22	Absolute frequencies are reported throughout as n/N (%), alongside ORs where relevant, including SBI frequency, death following documented neuro-WWLST, in-hospital death within SBI, severe-domain burden and survivor CP/cognitive outcome frequencies.
Results	17	Report other analyses done—e.g., analyses of subgroups and interactions, and sensitivity analyses	Main p. 14–20; Supplement p. 6–11; Supplement Tables S10–S26	Other analyses include entity-specific panels, scan-selection robustness, last-window/maximal-burden concordance, last available CUS comparisons, fixed CUS1–CUS2 analyses, within-entity modifiers, delayed-case interval change, CP motor phenotype, cognitive outcome categories and exploratory adjusted survivor outcome models.

Section	Item No.	Recommendation	Page No.	Relevant text from manuscript
**Discussion**	18	Summarize key results with reference to study objectives	Main p. 21 and Conclusions p. 25	The Discussion summarizes SBI burden, concentration of deaths following documented neuro-WWLST within SBI, distinct time-dependent serial CUS phenotypes, multidomain imaging burden, outcome-proximal last-window CUS and exploratory survivor outcomes.
Discussion	19	Discuss limitations, taking into account sources of potential bias or imprecision. Discuss both direction and magnitude of any potential bias	Main p. 24–25	Limitations include the retrospective single-center 30-year design, secular trends, routine report-based imaging, no systematic blinded re-review or interobserver reliability, non-uniform MRI availability, survival-dependent CUS-window availability, fatal-only neuro-WWLST ascertainment, sparse entity-specific event counts, restricted adjustment, heterogeneous cognitive outcome ascertainment and descriptive/non-causal interpretation.
Discussion	20	Give a cautious overall interpretation of results considering objectives, limitations, multiplicity, similar studies and other relevant evidence	Main p. 21–25	Interpretation is explicitly cautious: imaging findings are descriptive associations, not prediction or causal inference; severe multidomain burden indicates increased risk but is not deterministic; outcomes vary by SBI entity, motor phenotype and functional severity.

Section	Item No.	Recommendation	Page No.	Relevant text from manuscript
Discussion	21	Discuss the generalizability (external validity) of the study results	Main p. 25	Generalisability is discussed in relation to single-center design, variation in treatment-limitation practices and neurodevelopmental follow-up pathways across units and countries, and the need for multicenter studies using standardized CUS windows, three-domain definitions, explicit treatment-limitation ascertainment and harmonized outcomes.
**Other information**	22	Give the source of funding and the role of the funders	Main p. 26	The Funding section states: "This research received no external funding." No funder role was applicable.

Abbreviations: BW, birth weight; CP, cerebral palsy; CUS, cranial ultrasound; cPVL, cystic periventricular leukomalacia; GA, gestational age; GMFCS, Gross Motor Function Classification System; IVH, intraventricular hemorrhage; LP, parenchymal lesion pattern; MRI, magnetic resonance imaging; neuro-WWLST, withdrawal, withholding, or non-escalation of life-sustaining treatment because of poor neurological prognosis; PVHI, periventricular hemorrhagic infarction; SBI, severe brain injury; VM, ventriculomegaly.
